# Supplementary material for: Primary care service utilisation and outcomes in type 2 diabetes: a longitudinal cohort analysis
Source: BMJ Open. 2022 Jan 31;12(1):e054654. doi: 10.1136/bmjopen-2021-054654 (PMC8808402; doi:10.1136/bmjopen-2021-054654)
Supplement: Supplementary data [file bmjopen-2021-054654supp001.pdf]

Supplementary File 1: Table to show diagnostic read codes used to identify individuals with type 2 diabetes to include in analysis

**Read Code**	**Condition**
C10F.00	Type 2 diabetes mellitus
C10F000	Type 2 diabetes mellitus with renal complications
C10F011	Type II diabetes mellitus with renal complications
C10F100	Type 2 diabetes mellitus with ophthalmic complications
C10F.11	Type II diabetes mellitus
C10F111	Type II diabetes mellitus with ophthalmic complications
C10F200	Type 2 diabetes mellitus with neurological complications
C10F211	Type II diabetes mellitus with neurological complications
C10F300	Type 2 diabetes mellitus with multiple complications
C10F311	Type II diabetes mellitus with multiple complications
C10F400	Type 2 diabetes mellitus with ulcer
C10F411	Type II diabetes mellitus with ulcer
C10F500	Type 2 diabetes mellitus with gangrene
C10F511	Type II diabetes mellitus with gangrene
C10F600	Type 2 diabetes mellitus with retinopathy
C10F611	Type II diabetes mellitus with retinopathy
C10F700	Type 2 diabetes mellitus - poor control
C10F711	Type II diabetes mellitus - poor control
C10F800	Reaven's syndrome
C10F811	Metabolic syndrome X
C10F900	Type 2 diabetes mellitus without complication

C10F911	Type II diabetes mellitus without complication
C10FA00	Type 2 diabetes mellitus with mononeuropathy
C10FA11	Type II diabetes mellitus with mononeuropathy
C10FB00	Type 2 diabetes mellitus with polyneuropathy
C10FB11	Type II diabetes mellitus with polyneuropathy
C10FC00	Type 2 diabetes mellitus with nephropathy
C10FC11	Type II diabetes mellitus with nephropathy
C10FD00	Type 2 diabetes mellitus with hypoglycaemic coma
C10FD11	Type II diabetes mellitus with hypoglycaemic coma
C10FE00	Type 2 diabetes mellitus with diabetic cataract
C10FE11	Type II diabetes mellitus with diabetic cataract
C10FF00	Type 2 diabetes mellitus with peripheral angiopathy
C10FG00	Type 2 diabetes mellitus with arthropathy
C10FG11	Type II diabetes mellitus with arthropathy
C10FH00	Type 2 diabetes mellitus with neuropathic arthropathy
C10FJ00	Insulin treated Type 2 diabetes mellitus
C10FJ11	Insulin treated Type II diabetes mellitus
C10FK00	Hyperosmolar non-ketotic state in type 2 diabetes mellitus
C10FL00	Type 2 diabetes mellitus with persistent proteinuria
C10FL11	Type II diabetes mellitus with persistent proteinuria
C10FM00	Type 2 diabetes mellitus with persistent microalbuminuria
C10FM11	Type II diabetes mellitus with persistent microalbuminuria
C10FN00	Type 2 diabetes mellitus with ketoacidosis

C10FP00	Type 2 diabetes mellitus with ketoacidotic coma
C10FQ00	Type 2 diabetes mellitus with exudative maculopathy
C10FR00	Type 2 diabetes mellitus with gastroparesis
