# Supplementary material for: Primary care service utilisation and outcomes in type 2 diabetes: a longitudinal cohort analysis
Source: BMJ Open. 2022 Jan 31;12(1):e054654. doi: 10.1136/bmjopen-2021-054654 (PMC8808402; doi:10.1136/bmjopen-2021-054654)
Supplement: Author's manuscript [file bmjopen-2021-054654.draft_revisions.pdf]

BMJ Open is committed to open peer review. As part of this commitment we make the peer review history of every article we publish publicly available.

When an article is published we post the peer reviewers' comments and the authors' responses online. We also post the versions of the paper that were used during peer review. These are the versions that the peer review comments apply to.

The versions of the paper that follow are the versions that were submitted during the peer review process. They are not the versions of record or the final published versions. They should not be cited or distributed as the published version of this manuscript.

BMJ Open is an open access journal and the full, final, typeset and author-corrected version of record of the manuscript is available on our site with no access controls, subscription charges or pay-per-view fees (http://bmjopen.bmj.com).

If you have any questions on BMJ Open's open peer review process please email info.bmjopen@bmj.com

BMJ Open

Primary care service utilisation and outcomes in type 2 diabetes: A longitudinal cohort analysis

Journal:	*BMJ Open*
Manuscript ID	bmjopen-2021-054654
Article Type:	Original research
Date Submitted by the Author:	21-Jun-2021
Complete List of Authors:	Hodgson, Sam; University of Southampton, Primary Care Research Centre<br>Morgan-Harrisskitt, Jeffrey; NHS South Central and West Commissioning Support Unit<br>Hounkpatin, Hilda; University of Southampton, Primary Care Research Centre<br>Stuart, Beth; University of Southampton<br>Dambha-Miller, Hajira; University of Southampton, Primary Care Research Centre
Keywords:	PRIMARY CARE, General diabetes < DIABETES & ENDOCRINOLOGY, Organisation of health services < HEALTH SERVICES ADMINISTRATION & MANAGEMENT

SCHOLARONE™
Manuscripts

I, the Submitting Author has the right to grant and does grant on behalf of all authors of the Work (as defined in the below author licence), an exclusive licence and/or a non-exclusive licence for contributions from authors who are: i) UK Crown employees; ii) where BMJ has agreed a CC-BY licence shall apply, and/or iii) in accordance with the terms applicable for US Federal Government officers or employees acting as part of their official duties; on a worldwide, perpetual, irrevocable, royalty-free basis to BMJ Publishing Group Ltd ("BMJ") its licensees and where the relevant Journal is co-owned by BMJ to the co-owners of the Journal, to publish the Work in this journal and any other BMJ products and to exploit all rights, as set out in our licence.

The Submitting Author accepts and understands that any supply made under these terms is made by BMJ to the Submitting Author unless you are acting as an employee on behalf of your employer or a postgraduate student of an affiliated institution which is paying any applicable article publishing charge ("APC") for Open Access articles. Where the Submitting Author wishes to make the Work available on an Open Access basis (and intends to pay the relevant APC), the terms of reuse of such Open Access shall be governed by a Creative Commons licence – details of these licences and which Creative Commons licence will apply to this Work are set out in our licence referred to above.

Other than as permitted in any relevant BMJ Author's Self Archiving Policies, I confirm this Work has not been accepted for publication elsewhere, is not being considered for publication elsewhere and does not duplicate material already published. I confirm all authors consent to publication of this Work and authorise the granting of this licence.

For peer review only

Primary care service utilisation and outcomes in type 2 diabetes: A longitudinal cohort analysis

Sam Hodgson[1], Jeffrey Morgan-Harrisskitt[2], Hilda Hounkpatin[1], Beth Stuart[1] and Hajira Dambha-Miller[1]

1. Primary Care Research Centre, University of Southampton, UK
2. Strategy and Transformation Department, NHS South, Central and West, UK

Correspondence: Dr Sam Hodgson, Primary Care Research Centre, University of Southampton, Southampton, SO16 5ST, Email: S.c.b.hodgson@soton.ac.uk

Keywords: type 2 diabetes; primary care; service utilisation; stratification

Word count

Main text: 2505

Abstract: 289/300

For peer review only

Abstract

Objectives: To describe primary care utilisation patterns amongst adults with type 2 diabetes and to quantify the association between utilisation and long-term health outcomes.

Design: Retrospective cohort study

Setting: 168 primary care practices in Southern England within the Electronic Care and Health Information Analytics (CHIA) database between 2013 and 2020

Participants: 110,240 adults with Quality and Outcomes Framework (QOF) read code of type 2 diabetes diagnosis; age greater than 18 years; linked and continuous records available from April 2013 until April 2020 (or death).

Primary and secondary outcome measures: 1) rates of service utilisation (total number of primary care contacts per quarter) across the study period; 2) participant characteristics associated with higher and lower rates of service utilisation; 3) associations between service utilisation and: i) cardiovascular disease (CVD events), and ii) all-cause mortality.

Results: Mean (SD) number of primary care attendances per quarter in the cohort of 110,240 went from 2.49 (2.01) in 2013 to 2.78 (2.06) in 2020. Patients in the highest-usage tertile were more likely to be female, older, more frail, white, from the least deprived quintile, and to have 5 or more comorbidities. In adjusted models, higher rates of service utilisation (per consultation) were associated with higher rates of CVD events (odds ratio (OR) 1.0058; 95% CI 1.0053 – 1.0062; p<0.001) and mortality (OR 1.0057; 95% CI 1.0051 – 1.0064; p<0.001)

Conclusions: People with type 2 diabetes are using primary care services more frequently, but increased volume of clinical care does not correlate with better outcomes, although this finding may be driven by more unwell patients contacting services more frequently. Further research on the nature and content of contacts is required to understand how to tailor services to deliver effective care to those at greatest risk of complications.

Article Summary / Strengths and limitations of this study

- This study includes a large sample of 110,240 individuals with a relatively long follow-up period of seven years.
- This study analyses robust and precise measures of clinical parameters from real-world clinical data.
- The retrospective and observational nature of the study design limits permissible inferences on causality or directionality of associations.
- Our data do not capture information on the nature, quality, or duration of primary care contacts, nor the relevance of that contact to type 2 diabetes.
- Although the study population is large, it is taken from an area of the UK (Southern England) which is more affluent, less ethnically diverese, and less socioeconomically deprived than the UK average, which may limit generalisability of findings.

For peer review only

Introduction:

Diabetes UK estimate that by 2030 5.5 million individuals in the UK will be diagnosed with type 2 diabetes[1], which today costs the NHS over £10 billion a year.[2] Strategies to optimise treatment and prevent associated complications are therefore a public health priority. The UK Government's NHS long-term plan builds on the General Practice Forward View articulating the need for better prevention of cardiovascular (CVD) disease[3]. One key area of focus is the prevention of type 2 diabetes and its related CVD complications in primary care, where over 80% of the disease is managed. It is hypothesised that primary care contacts could provide opportunities to positively influence and support healthy behaviours, optimise treatment plans, promote self-management and detect ill-health earlier.

For this reason, the last few years have seen greater provision of primary care diabetes services through increased multidisciplinary teams, automated recall and greater efforts at monitoring of diabetes progress through the UK National Diabetes Audit[4], a national surveillance programme assessing the quality of type 2 diabetes care in UK primary care, and Quality Outcome Framework (QOF)[5]. However, both the QOF and National Diabetes Audit have demonstrated large variations in primary care diabetes uptake while long-term outcomes remain poor.[6,7] Type 2 diabetes is still the leading cause of premature mortality and for someone diagnosed at the age of 50 years, for example, average life expectancy in the UK is reduced by six years.[8] Despite increased research, funding and the development of a number of interventions to prevent diabetes complications, few have been effectively translated in primary care. [9–12] In part, this may reflect the fact that national strategies and previous diabetes research do not adequately consider the contextual or real-world challenges in primary care. To develop effective strategies that are relevant and applicable to this setting, it is necessary to first understand the current utilisation and availability of primary care services to understand the context in which any future interventions would be nested. [13]

These contextual issues are critical to examine at a time when primary care is rapidly adapting to increasing demands on services, a growing burden of chronic disease and challenges in workforce recruitment. Cuts to public health and social funding with no planned increases in capital, education, training and local authority public health or social care are likely to add to existing pressures. [14–17] Several studies have already described patterns of service utilisation, characteristics of service users, and how these relate to health outcomes in general terms, but these have not specifically considered type 2 diabetes in primary care,[18] nor long-term complications such as heart attacks and strokes that have the greatest impact of patient's lives and on healthcare costs. [19,20]

The availability of longitudinal electronic primary care health records that include large cohorts with objective measures provides an opportunity to overcome the limitations of previous research in this area and inform future provision. In this study, we examined patterns of primary care service utilisation in adults with type 2 diabetes in a large population-based cohort, and quantified the association between utilisation and long-term health outcomes.

Method:

Design

Retrospective cohort analysis

Data source

The Electronic Care and Health Information Analytics (CHIA) database includes individual level anonymised live data from primary care records linked to local acute hospital trusts in the South of England (Hampshire). Data from

For peer review only - http://bmjopen.bmj.com/site/about/guidelines.xhtml

1.5 million consenting patient medical records have been anonymised and collected continuously across 168 primary care practices covering urban and rural populations. Data includes coded clinical entries on demographic data, service utilisation, diagnoses, investigations, medications and clinical outcomes from primary care and hospital laboratories.

Study population

Individuals were included if they met the following criteria: age greater than 18 years; linked and continuous records available from April 2013 until April 2020 (or death); Quality and Outcomes Framework (QOF) read code of type 2 diabetes diagnosis ((including codes C10F.00, C10F.11, and complication-related derivatives; full list of included codes given in supplementary file 1).

Exposure

Primary care service utilisation was defined as the total number of contacts during the in-hours service over the study period which was between April 2013 and April 2020. This included telephone, in-person or home visits recorded in the electronic primary care record.

Outcomes

i) Cardiovascular (CVD) events, defined as a composite of myocardial infarction, amputation, and stroke
ii) All-cause mortality defined as death from any cause during the study period

Covariates

Baseline sociodemographic data: We extracted data on age, sex and ethnicity. Socioeconomic deprivation was determined by the index of multiple deprivation (IMD), a score calculated by the government to reflect deprivation in a specific geographic area determined based on 7 domains of deprivation including income, employment, education, health, and crime[21].

Baseline clinical variables: Baseline comorbidities were defined from diagnostic codes of existing QOF conditions and included coronary heart disease, chronic kidney disease, chronic obstructive pulmonary disease (COPD), asthma, cancer, dementia, atrial fibrillation, epilepsy, heart failure, stroke, peripheral vascular disease, hypertension, osteoporosis, osteoarthritis, depression and frailty. Frailty was defined using the Electronic Frailty Index Score, a validated tool used to estimate an individual's frailty state using data automatically held in primary care health records [22]. Latest smoking status (current, ex or never-smoked), weight, body mass index (BMI), systolic and diastolic blood pressure and biochemistry measures (including HbA_{1c} total cholesterol, HDL-cholesterol and eGF) were examined as the earliest available recordings from April 1st 2013

Duration of type 2 diabetes: We estimated the interval between the first code for type 2 diabetes in the electronic record until study entry (April 2013).

Statistical Analysis:

Baseline characteristics were summarised using descriptive statistics. Time series graphs were plotted using mean utilisation for each quarter over the 7-year follow-up. We compared these between sociodemographic variables. Service utilisation was summarised with descriptive statistics and divided into tertiles in line with previous approaches in the literature[23]; we tested for differences between tertiles using one-way analysis of variance for

continuous variables (ANOVA) and a χ^2-test for categorical variables. Finally, we constructed univariate and multivariable logistic models on a complete-case analysis to examine the association between service utilisation (total number of contacts over the study duration), CVD events, and all-cause mortality.

Multivariable logistic regression models were adjusted for potential confounders on *a priori* reasoning including baseline sociodemographic variables (age, sex, ethnicity and IMD); clinical variables to take into account disease severity as sicker patients might attend more frequently (HbA1c, blood pressure, total cholesterol, BMI, frailty, total number of medications prescribed and smoking status); and practice level co-variates (to account for clustering). Parameter estimates are presented with 95% confidence intervals. Tests were conducted as two-sided, at the 5% significance level. Data were analysed using STATA version 16.1 and R version 4.0.3. Our findings are reported in line with the STROBE and RECORD guidelines for observational studies using routinely collected health data[24].

Ethical Approval

The Hampshire Health Record (Care and Health Information Exchange) has its own independent panel for ethics and governance approval. We have been granted governance and ethics approval. We have additionally received approval from the University of Southampton Research and Governance office (submission reference: ERGO49232).

Patient and Public Involvement

No direct involvement.

<u>Results:</u>

Participant characteristics

The cohort included 110 240 people with type 2 diabetes across 168 primary care practices in Southern England. The mean (SD) age of participants at baseline was 62.89 (14.29) years, there were more men than women (55.82%), the majority were white (46.83%) and over a quarter of the cohort came from the highest socioeconomic quintile (IMD 5, 26.46%). These results are summarised in Table 1. We compared participants with and without missing data, with ethnicity having the largest number of missing data (48%), which is not unusual for routinely collected primary care data[25]. Participants with missing data were more likely to be male, white and from the highest socioeconomic quintile.

Primary care service utilization

Across the whole study period (April 2013 to April 2020), mean number of attendances per patient was 74.0 (2.68 per quarter, SD 2.04). Seasonal variations were observed within each year (with higher use in winter) but overall, mean (annual rolling average) utilisation for the cohort increased over the study period from 2.48 (2.01) per quarter in 2013 to 2.78 (2.06) attendances per quarter in 2020 (figure 1). This increase was observed across all age groups (figure 2A) and was highest amongst those aged 65-84 years. Attendance rates were higher in White patients compared to Black, Asian, or other ethnic minority backgrounds; (figure 2B).

Characteristics of patients by service utilisation tertile

Sociodemographic and clinical characteristics of included individuals are described by service utilisation tertile in table 2. Mean (SD) utilisation of primary care services was four times greater in the highest utilisation tertile (119.7 (47.1) attendances) compared to the lowest (38.6 (8.9) attendances)($p<0.001$). Patients in the highest-usage tertile were more likely to be female, older, more frail, white, from the least deprived IMD quintile, and to have 5 or more comorbidities. Patients in the lowest-usage tertile were more likely to be male, younger, non-White, less multimorbid, less frail, and from the most deprived IMD quintile. Mean (SD) HbA1c was higher in the highest utilisation tertile (60.2 (17.65) mmol/mol)) than the lowest utilisation tertile (57.9 (16.83) mmol/mol).

For peer review only - http://bmjopen.bmj.com/site/about/guidelines.xhtml

Primary care utilisation and outcomes

In both univariate and maximally adjusted models (table 3), higher rates of service utilisation were associated with higher rates of cardiovascular disease (adjusted OR 1.0057 per additional primary care attendance; 95% CI 1.0052 – 1.0061; p<0.001) and mortality (adjusted OR 1.0056; 95% CI 1.0050 – 1.0063; p<0.001).

Discussion

Summary of key findings

In this retrospective cohort study of 110,240 people with type 2 diabetes, we observed growing rates of primary care service utilisation between 2013 and 2020. This was highest amongst White females with multimorbidity and polypharmacy from higher socio-economic backgrounds. After adjusting for potential confounders including age and co-morbidities, we find more frequent contacts with primary care is associated with higher risk of CVD events and mortality. These findings could suggest a need to focus on personalisation of care, and quality of delivered care, to improve outcomes in type 2 diabetes; or could reflect reverse causality, ie, services are being accessed more frequently by those with greater health needs.

Strengths and Limitations

Strengths of our study include its large sample of 110,240 people and a relatively long follow-up period of seven years with robust and precise measures of clinical parameters, and restriction of analysis to complete cases. Limitations include the retrospective, observational nature of our study design which limits permissible inferences on causality or directionality of associations; it is unclear from our findings why a relationship between higher rates of service utilisation and poor outcomes exists in type 2 diabetes, and reverse causality may well underlie these findings, ie, more unwell patients requiring more frequent care and being invited for more regular reviews. Our data do not capture information on the nature, quality, or duration of primary care contacts; who the contact was with (eg a GP or specialist nurse); nor the relevance of that contact to type 2 diabetes; for example, patients attending once yearly for a 30 minute review of their type 2 diabetes would appear to be using services to a lesser extent than patients attending three times per year for 10 minute appointments with minor ailments, but may be receiving more focused, high-quality diabetes care. Although the study population is large and includes a large geographic area in the UK, it does include a more affluent, less ethnically diverse and less socioeconomically deprived population than the UK average, which may limit generalisability of findings. Relatively high rates of missing data on participant ethnicity are in keeping with typical results taken from real-world primary care databases, but this missing data is an important limitation to interpretation of findings related to ethnicity.

Comparison to existing literature

Our findings are in agreement with those of Hobbs et al describing trends of increasing primary care utilisation between 2007 – 2014 in England[18]. While most studies in this domain have examined trends in general utilisation of primary care, our study is novel in specifically examining patters of service use among individuals with type 2 diabetes. Our finding that increased service utilisation is not associated with better outcomes in type 2 diabetes suggests simple expansion of number of primary care contacts may not necessarily be beneficial, and that there is a need to consider the quality, nature and content of contacts when tailoring service design in type 2 diabetes.

In a recent cross-sectional observational study assessing routine primary care data from 2.7 million English patients, Lay-Flurrie et al report no association between service utilization and mortality[26]. In their analysis, the authors did not stratify by underlying diagnosis, and just 16.5% of included patients were aged over 65 years, in contrast to 50.4% in our cohort. These findings imply service utilisation might offer benefit as a predictor of poor outcomes in selected, but not unselected, primary care populations, such as adults with type 2 diabetes.

We find that in patients with type 2 diabetes higher numbers of primary care attendances were associated with a higher odds of cardiovascular events and mortality, even after taking into account co-morbidities and socio-

For peer review only - http://bmjopen.bmj.com/site/about/guidelines.xhtml
For peer review only

demographic factors. One explanation for this finding could be that optimal preventive care cannot always be achieved in the context of services poor in time and funding resources, despite high frequency contacts. This would be supported by findings from semi-structured interviews in patients with type 2 diabetes, GPs and specialist nurses, which find that only “minimum care standards can be maintained” in providing care to patients with type 2 diabetes[27].

Our study does not inform on the mechanisms underlying the associations between service utilisation, CVD and mortality. It is possible reverse causality[28]could underlie this finding: patients with established cardiovascular disease, for example, are more likely to use primary care services for medication reviews and routine care, and also more likely to develop further cardiovascular disease, or die[29]. Patients who are more frail and multimorbid, or with more difficult to manage or refractory disease, may also be more likely to use primary care services more frequently[30], or to be followed up more intensively by their clinicians, although we adjusted for these potential confounders in our analysis. Alternatively, this finding could reflect underlying mechanisms not captured in our adjusted models, such as a higher burden of non-cardiovascular diseases associated with mortality such as cancer and COPD [31].

Implications for Research and Practice

This study shows an increase of 8.3% in rates of primary care service utilization by patients with type 2 diabetes between 2013 and 2020. Highest rates of utilisation were seen in older and frailer patients. In the context of growing pressures on primary care services from increasingly complex patients, there is an urgent need to consider strategies to deliver high quality diabetes care in the community to meet growing demand, while acknowledging that simply increasing the capacity of services does not necessarily mean improved outcomes.

As opposed to achieving better disease control and developing fewer complications, patients with more frequent primary care contacts had higher HbA1c, higher rates of CVD, and higher rates of mortality. Although these patients tended to be older and more multimorbid, these associations persisted after adjustment for these and other confounders. Further research capturing the nature, content and duration of contacts in relation to delivery of care for type 2 diabetes is needed to understand the mechanisms underlying this association and address contributory factors.

Funding statement:

HDM is a National Institute for Health Research (NIHR) funded Academic Clinical Lecturer and has received NIHR School of Primary Care Research funding to support this work (SPCR2014-10043). SH is an Academic Clinical Fellow funded by the NIHR. The views and opinions expressed by authors in this publication are those of the authors and do not necessarily reflect those of the UK National Institute for Health Research (NIHR) or the Department of Health and Social Care.

Author contributions:

HDM designed the study, wrote the first draft of the paper, edited and contributed to subsequent versions. SH analysed the data, and drafted and revised subsequent versions of the paper. JH carried out data analysis and revised the paper. HH revised the paper. BS provided advice on statistical methods and revised the paper.

Competing Interests:

None to declare.

For peer review only - http://bmjopen.bmj.com/site/about/guidelines.xhtml

Data availability statement

Access to the dataset used for this research is governed by the Hampshire Health Record (Care and Health Information Exchange), which has its own independent panel for ethics and governance approval, and data access. More information on their procedures can be found here: https://careandhealthinformationexchange.org.uk/wp-content/uploads/2018/04/CHIE-Review-of-Procedures-Compliance-with-GDPR_V5-1.pdf.

References:

1 Diabetes UK. Us, diabetes and a lot of facts and stats. *Diabetes UK* 2019;:1–48.www.diabetes.org.uk (accessed 11 Feb 2021).

2 Hex N, Bartlett C, Wright D, *et al.* Estimating the current and future costs of Type1 and Type2 diabetes in the UK, including direct health costs and indirect societal and productivity costs. *Diabet Med* 2012;**29**:855–62. doi:10.1111/j.1464-5491.2012.03698.x

3 National Health Service. The NHS Long Term Plan. 2019. https://www.longtermplan.nhs.uk/publication/nhs-long-term-plan/ (accessed 14 Jan 2021).

4 Heald AH, Livingston M, Malipatil N, *et al.* Improving type 2 diabetes mellitus glycaemic outcomes is possible without spending more on medication: Lessons from the UK National Diabetes Audit. *Diabetes, Obes Metab* 2018;**20**:185–94. doi:10.1111/dom.13067

5 Forbes LJL, Marchand C, Doran T, *et al.* The role of the Quality and Outcomes Framework in the care of long-term conditions: A systematic review. *Br J Gen Pract* 2017;**67**:e775–84. doi:10.3399/bjgp17X693077

6 Gadsby R, Young B. Diabetes care in England and Wales: information from the 2010-2011 National Diabetes Audit. *Diabet Med* 2013;**30**:799–802. doi:10.1111/dme.12182

7 Wright CE, Yeung S, Knowles H, *et al.* Factors influencing variation in participation in the National Diabetes Audit and the impact on the Quality and Outcomes Framework indicators of diabetes care management. *BMJ Open Diabetes Res Care* 2018;**6**:e000554. doi:10.1136/bmjdrc-2018-000554

8 International Diabetes Federation. IDF Diabetes Atlas, 7th edn. Brussels, Belgium: International Diabetes Federation 2015. *International Diabetes Federation. IDF Diabetes Atlas, 7th edn. Brussels, Belgium: International Diabetes Federation, 2015.*

9 Mc Hugh S, O'Mullane M, Perry IJ, *et al.* Barriers to, and facilitators in, introducing integrated diabetes care in Ireland: a qualitative study of views in general practice. *BMJ Open* 2013;**3**:e003217. doi:10.1136/bmjopen-2013-003217

10 Kutz TL, Roszhart JM, Hale M, *et al.* Improving comprehensive care for patients with diabetes. *BMJ Open Qual* 2018;**7**:e000101. doi:10.1136/bmjoq-2017-000101

11 Bongaerts BWC, Müssig K, Wens J, *et al.* Effectiveness of chronic care models for the management of type 2 diabetes mellitus in Europe: a systematic review and meta-analysis. *BMJ Open* 2017;**7**:e013076. doi:10.1136/BMJOPEN-2016-013076

12 Lim LL, Lau ESH, Kong APS, *et al.* Aspects of Multicomponent Integrated Care Promote Sustained Improvement in Surrogate Clinical Outcomes: A Systematic Review and Meta-analysis. *Diabetes Care* 2018;**41**:1312–20. doi:10.2337/dc17-2010

For peer review only - http://bmjopen.bmj.com/site/about/guidelines.xhtml

13 Dambha-Miller H, Cooper AJM, Kinmonth AL, *et al.* Effect on cardiovascular disease risk factors of interventions to alter consultations between practitioners and patients with type 2 diabetes: A systematic review and meta-analysis of trials in primary care. *Heal Expect* Published Online First: February 2017. doi:10.1111/hex.12546

14 Roland M, Everington S. Tackling the crisis in general practice. *BMJ* 2016;**352**:i942. doi:10.1136/BMJ.I942

15 The King's Fund. Public satisfaction with the NHS and social care in 2017. 2017.

16 Maruthappu M, Sood HS, Keogh B. The NHS Five Year Forward View: implications for clinicians. *BMJ* 2014;**349**:g6518.

17 The King's Fund. The NHS long-term plan explained. 2019.

18 Hobbs FDR, Bankhead C, Mukhtar T, *et al.* Clinical workload in UK primary care: a retrospective analysis of 100 million consultations in England, 2007–14. *Lancet* 2016;**387**:2323–30. doi:10.1016/S0140-6736(16)00620-6

19 L.J. H, T.B. D, Renders CM, *et al.* Interventions to improve the management of diabetes mellitus in primary health care and outpatient community settings. L.J. Hansen, Central Forskningsenhed for Almen Praksis og Afdeling for Almen Medicin, Panum Instituttet, Kobenhavns Universitet, DK-2200 Kobenhavn N.: : John Wiley & Sons, Ltd 2002. doi:10.1002/14651858.CD001481

20 Griffin SJ, Kinmonth A-L, Veltman MWM, *et al.* Effect on health-related outcomes of interventions to alter the interaction between patients and practitioners: a systematic review of trials. *Ann Fam Med* 2004;**2**:595–608. doi:10.1370/afm.142

21 Jordan H, Roderick P, Martin D. The Index of Multiple Deprivation 2000 and accessibility effects on health. *J Epidemiol Community Health* 2004;**58**:250–7. doi:10.1136/jech.2003.013011

22 Clegg A, Bates C, Young J, *et al.* Development and validation of an electronic frailty index using routine primary care electronic health record data. *Age Ageing* 2016;**45**:353–60. doi:10.1093/ageing/afw039

23 Turrell G, Oldenburg BF, Harris E, *et al.* Utilisation of general practitioner services by socio-economic disadvantage and geographic remoteness. *Aust N Z J Public Health* 2004;**28**:152–8. doi:10.1111/j.1467-842X.2004.tb00929.x

24 Benchimol EI, Smeeth L, Guttmann A, *et al.* The REporting of studies Conducted using Observational Routinely-collected health Data (RECORD) Statement. *PLoS Med* 2015;**12**:1001885. doi:10.1371/journal.pmed.1001885

25 Mathur R, Bhaskaran K, Chaturvedi N, *et al.* Completeness and usability of ethnicity data in UK-based primary care and hospital databases. *J Public Heal (United Kingdom)* 2014;**36**:684–92. doi:10.1093/pubmed/fdt116

26 Lay-Flurrie S, Mathieu E, Bankhead C, *et al.* Patient consultation rate and clinical and NHS outcomes: A cross-sectional analysis of English primary care data from 2.7 million patients in 238 practices. *BMC Health Serv Res* 2019;**19**. doi:10.1186/s12913-019-4036-y

27 Dambha-Miller H, Griffin SJ, Kinmonth AL, *et al.* Provision of services in primary care for type 2 diabetes: a qualitative study with patients, GPs, and nurses in the East of England. *Br J Gen Pract* 2020;**70**:E668–75. doi:10.3399/bjgp20X710945

For peer review only

For peer review only - http://bmjopen.bmj.com/site/about/guidelines.xhtml

28 Leszczensky L, Wolbring T. How to Deal With Reverse Causality Using Panel Data? Recommendations for Researchers Based on a Simulation Study. *Sociol Methods Res* 2019;:004912411988247. doi:10.1177/0049124119882473

29 Damen JAAG, Hooft L, Schuit E, *et al.* Prediction models for cardiovascular disease risk in the general population: Systematic review. BMJ. 2016;**353**:i2416. doi:10.1136/bmj.i2416

30 Nunes BP, Flores TR, Mielke GI, *et al.* Multimorbidity and mortality in older adults: A systematic review and meta-analysis. Arch. Gerontol. Geriatr. 2016;**67**:130–8. doi:10.1016/j.archger.2016.07.008

31 Burney P, Jithoo A, Kato B, *et al.* Chronic obstructive pulmonary disease mortality and prevalence: The associations with smoking and poverty-A bold analysis. *Thorax* 2014;**69**:465–73. doi:10.1136/thoraxjnl-2013-204460

For peer review only

Table 1: Sociodemographic and clinical characteristics of the CHIA type 2 diabetes cohort

Population Sample	
Population Sample	n= 110240
Sex	
Female n(%)	48706 (44.18)
Male n(%)	61534 (55.82)
Patients Missing Data n(%)	0 (0)
Age (years)	
Age mean(SD)	62.89 (14.29)
Patients Missing Data n(%)	0 (0)
Ethnicity	
Asian n(%)	3295 (2.99)
Black n(%)	498 (0.45)
Mixed or Other n(%)	810 (0.73)
White n(%)	51626 (46.83)
Patients Missing Data n(%)	54011 (48.99)
Index of Multiple Deprivation	
1 n(%)	14035 (12.73)
2 n(%)	21833 (19.8)
3 n(%)	21064 (19.11)
4 n(%)	23062 (20.92)
5 n(%)	29171 (26.46)
Patients Missing Data n(%)	1075 (0.98)
Smoking Status	
Current Smoker n(%)	13875 (12.59)
Ex Smoker n(%)	44875 (40.71)
Never Smoked n(%)	50998 (46.26)
Patients Missing Data n(%)	492 (0.45)

For peer review only - http://bmjopen.bmj.com/site/about/guidelines.xhtml

**Total co-morbidities**	
Total co-morbidities mean(SD)	2.36 (1.79)
Patients Missing Data n(%)	0 (0)
**Individual co-morbidities**	
Stroke n(%)	7694 (6.98)
Patients Missing Data n(%)	0 (0)
Myocardial Infarction n(%)	9812 (8.9)
Patients Missing Data n(%)	0 (0)
Peripheral Vascular Disease n(%)	3565 (3.23)
Patients Missing Data n(%)	0 (0)
Chronic Kidney Disease n(%)	15471 (14.03)
Patients Missing Data n(%)	0 (0)
Dementia n(%)	3558 (3.23)
Patients Missing Data n(%)	0 (0)
Total Medications mean(SD)	48.59 (30.12)
Patients Missing Data n(%)	0 (0)
Frailty Score mean(SD)	0.22 (0.11)
Patients Missing Data n(%)	77 (0.07)

Values are presented as mean (standard deviation) unless otherwise stated, all values are taken at baseline. HbA_{1c} = glycated haemoglobin

For peer review only

Table 2: Sociodemographic and clinical characteristics of the CHIA type 2 diabetes cohort presented by primary care utilisation tertiles

Variables	All Participants	Tertile 1 (low usage)	Tertile 2	Tertile 3 (high usage)	p-value
Complete case cohort					
Number of patients (n)	75701 (0)	25234 (0)	25234 (0)	25233 (0)	
Average attendances mean (SD)	74.04 (44)	38.6 (8.49)	63.8 (7.87)	119.73 (47.17)	<0.01
Sex					
Female n(%)	34887 (46.09)	9959 (39.47)	11739 (46.52)	13189 (52.27)	<0.01
Male n(%)	40814 (53.91)	15275 (60.53)	13495 (53.48)	12044 (47.73)	<0.01
Age at start (years)					
Age mean(SD)	63.15 (13.04)	60.2 (12.78)	63.05 (12.77)	66.19 (12.87)	<0.01
Age group at start (years)					
18-44 years n(%)	6506 (8.59)	2770 (10.98)	2102 (8.33)	1634 (6.48)	<0.01
45-64 years n(%)	31054 (41.02)	12436 (49.28)	10491 (41.57)	8127 (32.21)	<0.01
65-84 years n(%)	35804 (47.3)	9510 (37.69)	11931 (47.28)	14363 (56.92)	<0.01
85+ years n(%)	2337 (3.09)	518 (2.05)	710 (2.81)	1109 (4.4)	<0.01
Ethnicity					
Asian n(%)	2097 (2.77)	730 (2.89)	841 (3.33)	526 (2.08)	<0.01
Black n(%)	309 (0.41)	110 (0.44)	118 (0.47)	81 (0.32)	0.03
Mixed or Other n(%)	544 (0.72)	195 (0.77)	182 (0.72)	167 (0.66)	0.34
White n(%)	39519 (52.2)	11272 (44.67)	13543 (53.67)	14704 (58.27)	<0.01
Missing Data n(%)	33232 (43.9)	12927 (51.23)	10550 (41.81)	9755 (38.66)	<0.01
Index of Multiple Deprivation Quintile					
1 n(%)	9301 (12.29)	3311 (13.12)	3013 (11.94)	2977 (11.8)	<0.01
2 n(%)	14215 (18.78)	4857 (19.25)	4621 (18.31)	4737 (18.77)	0.01
3 n(%)	13924 (18.39)	4889 (19.37)	4477 (17.74)	4558 (18.06)	0.56
4 n(%)	16285 (21.51)	5415 (21.46)	5560 (22.03)	5310 (21.04)	0.01
5 n(%)	21368 (28.23)	6612 (26.2)	7348 (29.12)	7408 (29.36)	0.03

Missing IMD Values n(%)	608 (0.8)	150 (0.59)	215 (0.85)	243 (0.96)	<0.01
HbA1c (%)					
HbA1c mean(SD)	7.55 (1.53)	7.43 (1.48)	7.51 (1.49)	7.65 (1.59)	<0.01
HbA1c (mmol/mol)					
HbA1c mean(SD)	59.1 (16.97)	57.85 (16.38)	58.73 (16.49)	60.21 (17.65)	<0.01
Weight (kg)					
Weight mean(SD)	90.16 (21.7)	89.74 (20.88)	89.7 (21.11)	90.8 (22.67)	<0.01
Electronic frailty index score					
Mean Electronic frailty index score (SD)	0.22 (0.10)	0.16 (0.08)	0.22 (0.09)	0.30 (0.11)	<0.001
Number of co-morbidities					
<= 2 n(%)	40400 (53.37)	18048 (71.52)	13659 (54.13)	8693 (34.45)	<0.01
3-4 n(%)	23970 (31.66)	5996 (23.76)	8575 (33.98)	9399 (37.25)	<0.01
>=5 n(%)	11331 (14.97)	1190 (4.72)	3000 (11.89)	7141 (28.3)	<0.01
Total number of medications prescribed					
<=2 n(%)	1078 (1.42)	737 (2.92)	235 (0.93)	106 (0.42)	<0.01
3-5 n(%)	1037 (1.37)	674 (2.67)	285 (1.13)	78 (0.31)	<0.01
6-9 n(%)	1498 (1.98)	893 (3.54)	419 (1.66)	186 (0.74)	<0.01
>=10 n(%)	72088 (95.23)	22930 (90.87)	24295 (96.28)	24863 (98.53)	<0.01

Data sourced from CHIA dataset Values are presented as mean (standard deviation) unless otherwise stated, all values are taken at baseline. BMI = body mass index, HbA_{1c} = glycated haemoglobin)

For peer review only - http://bmjopen.bmj.com/site/about/guidelines.xhtml

Table 3: Univariate (unadjusted) and multivariate (adjusted) models describing the association between primary care service utilisation, defined as number of contacts with primary care services, cardiovascular disease and mortality, in the CHIA data set between 2013 and 2020

	**Unadjusted odds ratio (95% CI) per additional primary care contact**	**Univariate P Value**	**Adjusted odds ratio (95% CI) per additional primary care contact**	**Multivariate P value**
**Cardiovascular disease**	1.0071 (1.0066 - 1.0074)	<0.001	1.0057 (1.0052 - 1.0061)	<0.001
**Mortality**	1.0077 (1.0070 - 1.0082)	<0.001	1.0056 (1.0050 - 1.0063)	<0.001

Odds ratios describe the additional odds of outcome associated with each additional primary care contact. Models were adjusted for age, sex, ethnicity, index of multiple deprivation, smoking status, number of prescribed medications, frailty, BMI, HbA1c, systolic and diastolic blood pressure, mean total cholesterol, and practice-level co-morbidity prevalence to account for local clustering.

For peer review only

Figure 1: Mean number of primary care attendances by year quarter between 2013 and 2020 in the CHIA type 2 diabetes cohort. Q1 is defined as January – March.

Figure 2: Rolling four-quarter mean number of primary care attendances by year quarter between 2013 and 2020 in the CHIA type 2 diabetes cohort stratified by (A) age band (in years) at study entry and (B) ethnicity. Q1 is defined as January – March. For example, the mean number of attendances for Q1 2014 represents mean attendance between Q1 2013 and Q1 2014.

For peer review only

For peer review only - http://bmjopen.bmj.com/site/about/guidelines.xhtml

For peer review only

For peer review only - http://bmjopen.bmj.com/site/about/guidelines.xhtml

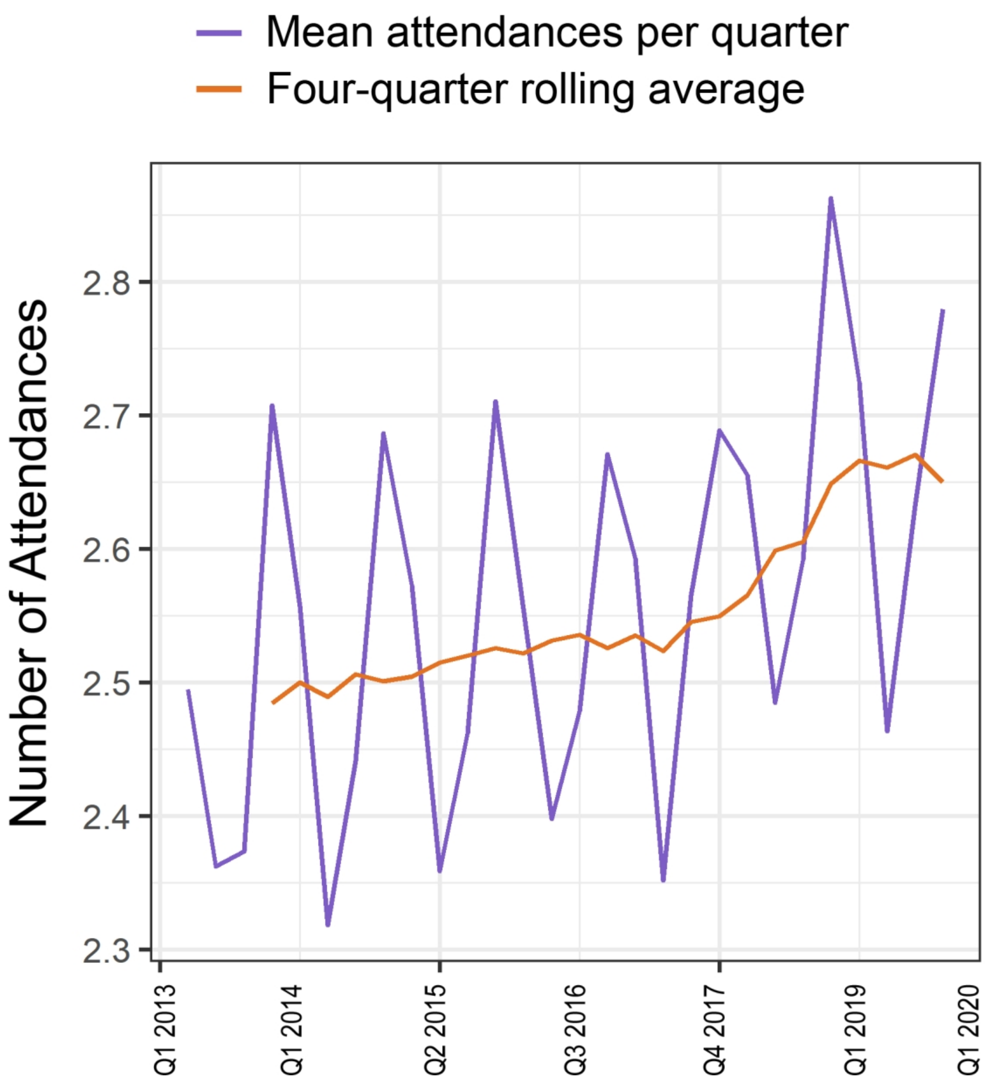

Figure 1: Mean number of primary care attendances by year quarter between 2013 and 2020 in the CHIA type 2 diabetes cohort. Q1 is defined as January – March.

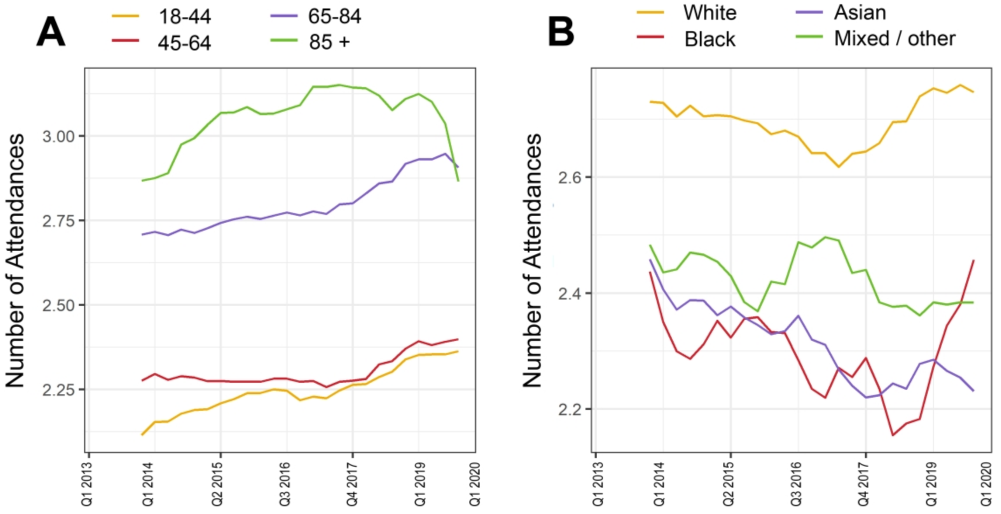

Figure 2: Rolling four-quarter mean number of primary care attendances by year quarter between 2013 and 2020 in the CHIA type 2 diabetes cohort stratified by (A) age band (in years) at study entry and (B) ethnicity. Q1 is defined as January – March. For example, the mean number of attendances for Q1 2014 represents mean attendance between Q1 2013 and Q1 2014.

Supplementary File 1: Table to show diagnostic read codes used to identify individuals with type 2 diabetes to include in analysis

Read Code	Condition
C10F.00	Type 2 diabetes mellitus
C10F000	Type 2 diabetes mellitus with renal complications
C10F011	Type II diabetes mellitus with renal complications
C10F100	Type 2 diabetes mellitus with ophthalmic complications
C10F.11	Type II diabetes mellitus
C10F111	Type II diabetes mellitus with ophthalmic complications
C10F200	Type 2 diabetes mellitus with neurological complications
C10F211	Type II diabetes mellitus with neurological complications
C10F300	Type 2 diabetes mellitus with multiple complications
C10F311	Type II diabetes mellitus with multiple complications
C10F400	Type 2 diabetes mellitus with ulcer
C10F411	Type II diabetes mellitus with ulcer
C10F500	Type 2 diabetes mellitus with gangrene
C10F511	Type II diabetes mellitus with gangrene
C10F600	Type 2 diabetes mellitus with retinopathy
C10F611	Type II diabetes mellitus with retinopathy
C10F700	Type 2 diabetes mellitus - poor control
C10F711	Type II diabetes mellitus - poor control
C10F800	Reaven's syndrome
C10F811	Metabolic syndrome X
C10F900	Type 2 diabetes mellitus without complication

For peer review only

C10F911	Type II diabetes mellitus without complication
C10FA00	Type 2 diabetes mellitus with mononeuropathy
C10FA11	Type II diabetes mellitus with mononeuropathy
C10FB00	Type 2 diabetes mellitus with polyneuropathy
C10FB11	Type II diabetes mellitus with polyneuropathy
C10FC00	Type 2 diabetes mellitus with nephropathy
C10FC11	Type II diabetes mellitus with nephropathy
C10FD00	Type 2 diabetes mellitus with hypoglycaemic coma
C10FD11	Type II diabetes mellitus with hypoglycaemic coma
C10FE00	Type 2 diabetes mellitus with diabetic cataract
C10FE11	Type II diabetes mellitus with diabetic cataract
C10FF00	Type 2 diabetes mellitus with peripheral angiopathy
C10FG00	Type 2 diabetes mellitus with arthropathy
C10FG11	Type II diabetes mellitus with arthropathy
C10FH00	Type 2 diabetes mellitus with neuropathic arthropathy
C10FJ00	Insulin treated Type 2 diabetes mellitus
C10FJ11	Insulin treated Type II diabetes mellitus
C10FK00	Hyperosmolar non-ketotic state in type 2 diabetes mellitus
C10FL00	Type 2 diabetes mellitus with persistent proteinuria
C10FL11	Type II diabetes mellitus with persistent proteinuria
C10FM00	Type 2 diabetes mellitus with persistent microalbuminuria
C10FM11	Type II diabetes mellitus with persistent microalbuminuria
C10FN00	Type 2 diabetes mellitus with ketoacidosis

For peer review only

C10FP00	Type 2 diabetes mellitus with ketoacidotic coma
C10FQ00	Type 2 diabetes mellitus with exudative maculopathy
C10FR00	Type 2 diabetes mellitus with gastroparesis

Supplementary File 1: Table to show diagnostic read codes used to identify individuals with type 2 diabetes to include in analysis

For peer review only

STROBE Statement—checklist of items that should be included in reports of observational studies

	Item No	Recommendation
**Title and abstract**	1	(*a*) Indicate the study's design with a commonly used term in the title or the abstract
		(*b*) Provide in the abstract an informative and balanced summary of what was done and what was found
**Introduction**		
Background/rationale	2	Explain the scientific background and rationale for the investigation being reported
Objectives	3	State specific objectives, including any prespecified hypotheses
**Methods**		
Study design	4	Present key elements of study design early in the paper
Setting	5	Describe the setting, locations, and relevant dates, including periods of recruitment, exposure, follow-up, and data collection
Participants	6	(*a*) *Cohort study*—Give the eligibility criteria, and the sources and methods of selection of participants. Describe methods of follow-up<br>*Case-control study*—Give the eligibility criteria, and the sources and methods of case ascertainment and control selection. Give the rationale for the choice of cases and controls<br>*Cross-sectional study*—Give the eligibility criteria, and the sources and methods of selection of participants
		(*b*) *Cohort study*—For matched studies, give matching criteria and number of exposed and unexposed<br>*Case-control study*—For matched studies, give matching criteria and the number of controls per case
Variables	7	Clearly define all outcomes, exposures, predictors, potential confounders, and effect modifiers. Give diagnostic criteria, if applicable
Data sources/ measurement	8*	For each variable of interest, give sources of data and details of methods of assessment (measurement). Describe comparability of assessment methods if there is more than one group
Bias	9	Describe any efforts to address potential sources of bias
Study size	10	Explain how the study size was arrived at
Quantitative variables	11	Explain how quantitative variables were handled in the analyses. If applicable, describe which groupings were chosen and why
Statistical methods	12	(*a*) Describe all statistical methods, including those used to control for confounding
		(*b*) Describe any methods used to examine subgroups and interactions
		(*c*) Explain how missing data were addressed
		(*d*) *Cohort study*—If applicable, explain how loss to follow-up was addressed<br>*Case-control study*—If applicable, explain how matching of cases and controls was addressed<br>*Cross-sectional study*—If applicable, describe analytical methods taking account of sampling strategy
		(*e*) Describe any sensitivity analyses

Continued on next page

For peer review only - http://bmjopen.bmj.com/site/about/guidelines.xhtml

**Results**		
Participants	13*	(a) Report numbers of individuals at each stage of study—eg numbers potentially eligible, examined for eligibility, confirmed eligible, included in the study, completing follow-up, and analysed
		(b) Give reasons for non-participation at each stage
		(c) Consider use of a flow diagram
Descriptive data	14*	(a) Give characteristics of study participants (eg demographic, clinical, social) and information on exposures and potential confounders
		(b) Indicate number of participants with missing data for each variable of interest
		(c) *Cohort study*—Summarise follow-up time (eg, average and total amount)
Outcome data	15*	*Cohort study*—Report numbers of outcome events or summary measures over time
		Case-control study—Report numbers in each exposure category, or summary measures of exposure
		Cross-sectional study—Report numbers of outcome events or summary measures
Main results	16	(*a*) Give unadjusted estimates and, if applicable, confounder-adjusted estimates and their precision (eg, 95% confidence interval). Make clear which confounders were adjusted for and why they were included
		(*b*) Report category boundaries when continuous variables were categorized
		(*c*) If relevant, consider translating estimates of relative risk into absolute risk for a meaningful time period
Other analyses	17	Report other analyses done—eg analyses of subgroups and interactions, and sensitivity analyses
**Discussion**		
Key results	18	Summarise key results with reference to study objectives
Limitations	19	Discuss limitations of the study, taking into account sources of potential bias or imprecision. Discuss both direction and magnitude of any potential bias
Interpretation	20	Give a cautious overall interpretation of results considering objectives, limitations, multiplicity of analyses, results from similar studies, and other relevant evidence
Generalisability	21	Discuss the generalisability (external validity) of the study results
**Other information**		
Funding	22	Give the source of funding and the role of the funders for the present study and, if applicable, for the original study on which the present article is based

*Give information separately for cases and controls in case-control studies and, if applicable, for exposed and unexposed groups in cohort and cross-sectional studies.

Note: An Explanation and Elaboration article discusses each checklist item and gives methodological background and published examples of transparent reporting. The STROBE checklist is best used in conjunction with this article (freely available on the Web sites of PLoS Medicine at http://www.plosmedicine.org/, Annals of Internal Medicine at http://www.annals.org/, and Epidemiology at http://www.epidem.com/). Information on the STROBE Initiative is available at www.strobe-statement.org.

For peer review only

For peer review only - http://bmjopen.bmj.com/site/about/guidelines.xhtml

Primary care service utilisation and outcomes in type 2 diabetes: A longitudinal cohort analysis

Journal:	*BMJ Open*
Manuscript ID	bmjopen-2021-054654.R1
Article Type:	Original research
Date Submitted by the Author:	20-Dec-2021

Complete List of Authors:	Hodgson, Sam; University of Southampton, Primary Care Research Centre<br>Morgan-Harrisskitt, Jeffrey; NHS South Central and West Commissioning Support Unit<br>Hounkpatin, Hilda; University of Southampton, Primary Care Research Centre<br>Stuart, Beth; University of Southampton<br>Dambha-Miller, Hajira; University of Southampton, Primary Care Research Centre

**Primary Subject Heading**:	Diabetes and endocrinology
Secondary Subject Heading:	General practice / Family practice
Keywords:	PRIMARY CARE, General diabetes < DIABETES & ENDOCRINOLOGY, Organisation of health services < HEALTH SERVICES ADMINISTRATION & MANAGEMENT

Note: The following files were submitted by the author for peer review, but cannot be converted to PDF. You must view these files (e.g. movies) online.
Fig 2AB.tif

I, the Submitting Author has the right to grant and does grant on behalf of all authors of the Work (as defined in the below author licence), an exclusive licence and/or a non-exclusive licence for contributions from authors who are: i) UK Crown employees; ii) where BMJ has agreed a CC-BY licence shall apply, and/or iii) in accordance with the terms applicable for US Federal Government officers or employees acting as part of their official duties; on a worldwide, perpetual, irrevocable, royalty-free basis to BMJ Publishing Group Ltd ("BMJ") its licensees and where the relevant Journal is co-owned by BMJ to the co-owners of the Journal, to publish the Work in this journal and any other BMJ products and to exploit all rights, as set out in our licence.

The Submitting Author accepts and understands that any supply made under these terms is made by BMJ to the Submitting Author unless you are acting as an employee on behalf of your employer or a postgraduate student of an affiliated institution which is paying any applicable article publishing charge ("APC") for Open Access articles. Where the Submitting Author wishes to make the Work available on an Open Access basis (and intends to pay the relevant APC), the terms of reuse of such Open Access shall be governed by a Creative Commons licence – details of these licences and which Creative Commons licence will apply to this Work are set out in our licence referred to above.

Other than as permitted in any relevant BMJ Author's Self Archiving Policies, I confirm this Work has not been accepted for publication elsewhere, is not being considered for publication elsewhere and does not duplicate material already published. I confirm all authors consent to publication of this Work and authorise the granting of this licence.

For peer review only

Primary care service utilisation and outcomes in type 2 diabetes: A longitudinal cohort analysis

Sam Hodgson[1], Jeffrey Morgan-Harrisskitt[2], Hilda Hounkpatin[1], Beth Stuart[1] and Hajira Dambha-Miller[1]

1. Primary Care Research Centre, University of Southampton, UK
2. Strategy and Transformation Department, NHS South, Central and West, UK

Correspondence: Dr Sam Hodgson, Primary Care Research Centre, University of Southampton, Southampton, SO16 5ST, Email: S.c.b.hodgson@soton.ac.uk / samcbhodgson@gmail.com

Keywords: type 2 diabetes; primary care; service utilisation; stratification

Word count

Main text: 2505

Abstract: 289/300

For peer review only

Abstract

Objectives: To describe primary care utilisation patterns amongst adults with type 2 diabetes and to quantify the association between utilisation and long-term health outcomes.

Design: Retrospective cohort study

Setting: 168 primary care practices in Southern England within the Electronic Care and Health Information Analytics (CHIA) database between 2013 and 2020

Participants: 110,240 adults with Quality and Outcomes Framework read code of type 2 diabetes diagnosis; age greater than 18 years; linked and continuous records available from April 2013 until April 2020 (or death).

Primary and secondary outcome measures: 1) rates of service utilisation (total number of primary care contacts per quarter) across the study period; 2) participant characteristics associated with higher and lower rates of service utilisation; 3) associations between service utilisation and: i) cardiovascular disease (CVD events), and ii) all-cause mortality.

Results: Mean (standard deviation) number of primary care attendances per quarter in the cohort of 110,240 went from 2.49 (2.01) in 2013 to 2.78 (2.06) in 2020. Patients in the highest-usage tertile were more likely to be female, older, more frail, white, from the least deprived quintile, and to have 5 or more comorbidities. In adjusted models, higher rates of service utilisation (per consultation) were associated with higher rates of CVD events (odds ratio (OR) 1.0058; 95% CI 1.0053 – 1.0062; p<0.001) and mortality (OR 1.0057; 95% CI 1.0051 – 1.0064; p<0.001)

Conclusions: People with type 2 diabetes are using primary care services more frequently, but increased volume of clinical care does not correlate with better outcomes, although this finding may be driven by more unwell patients contacting services more frequently. Further research on the nature and content of contacts is required to understand how to tailor services to deliver effective care to those at greatest risk of complications.

Article Summary / Strengths and limitations of this study

- This study includes a large sample of 110,240 individuals with a relatively long follow-up period of seven years.
- This study analyses robust and precise measures of clinical parameters from real-world clinical data.
- The retrospective and observational nature of the study design limits permissible inferences on causality or directionality of associations.
- Our data do not capture information on the nature, quality, or duration of primary care contacts, nor the relevance of that contact to type 2 diabetes.
- Although the study population is large, it is taken from an area of the UK (Southern England) which is more affluent, less ethnically diverese, and less socioeconomically deprived than the UK average, which may limit generalisability of findings.

For peer review only - http://bmjopen.bmj.com/site/about/guidelines.xhtml

Introduction:

Diabetes UK estimate that by 2030 5.5 million individuals in the United Kingdom (UK) will be diagnosed with type 2 diabetes[1], which today costs the National Health Service (NHS) over £10 billion a year.[2] Strategies to optimise treatment and prevent associated complications are therefore a public health priority. The UK Government's NHS long-term plan builds on the General Practice Forward View articulating the need for better prevention of cardiovascular disease (CVD) [3]. One key area of focus is the prevention of type 2 diabetes and its related CVD complications in primary care, where over 80% of the disease is managed[4]. It is hypothesised that primary care contacts could provide opportunities to positively influence and support healthy behaviours, optimise treatment plans, promote self-management and detect ill-health earlier.

For this reason, the last few years have seen greater provision of primary care diabetes services through increased multidisciplinary teams, automated recall and greater efforts at monitoring of diabetes progress through the UK National Diabetes Audit[5], a national surveillance programme assessing the quality of type 2 diabetes care in UK primary care, and Quality Outcome Framework (QOF)[6]. However, both the QOF and National Diabetes Audit have demonstrated large variations in primary care diabetes uptake while long-term outcomes remain poor.[7,8] Type 2 diabetes is still the leading cause of premature mortality and for someone diagnosed at the age of 50 years, for example, average life expectancy in the UK is reduced by six years.[9] Despite increased research, funding and the development of a number of interventions to prevent diabetes complications, few have been effectively translated in primary care. [10–13] In part, this may reflect the fact that national strategies and previous diabetes research do not adequately consider the contextual or real-world challenges in primary care. To develop effective strategies that are relevant and applicable to this setting, it is necessary to first understand the current utilisation and availability of primary care services to understand the context in which any future interventions would be nested. [14]

These contextual issues are critical to examine at a time when primary care is rapidly adapting to increasing demands on services, a growing burden of chronic disease and challenges in workforce recruitment. Cuts to public health and social funding with no planned increases in capital, education, training and local authority public health or social care are likely to add to existing pressures. [15–18] Several studies have already described patterns of service utilisation, characteristics of service users, and how these relate to health outcomes in general terms, but these have not specifically considered type 2 diabetes in primary care,[19] nor long-term complications such as heart attacks and strokes that have the greatest impact of patient's lives and on healthcare costs. [20,21]

The availability of longitudinal electronic primary care health records that include large cohorts with objective measures provides an opportunity to overcome the limitations of previous research in this area and inform future provision. In this study, we examined patterns of primary care service utilisation in adults with type 2 diabetes in a large population-based cohort, and quantified the association between utilisation and long-term health outcomes.

Method:

Design

Retrospective cohort analysis

Data source

The Electronic Care and Health Information Analytics (CHIA) database includes individual level anonymised live data from primary care records linked to local acute hospital trusts in the South of England (Hampshire). Data from

For peer review only - http://bmjopen.bmj.com/site/about/guidelines.xhtml

1.5 million consenting patient medical records have been anonymised and collected continuously across 168 primary care practices covering urban and rural populations. Patients automatically "opt in" to their anonymised data being included in the research data set, and can opt out via their primary care provider or the CHIE platform. Data on the proportion of patients opting out was not available to the research team. Data includes coded clinical entries on demographic data, service utilisation, diagnoses, investigations, medications and clinical outcomes from primary care and hospital laboratories.

Study population

Individuals were included if they met the following criteria: age greater than 18 years; linked and continuous records available from April 2013 until April 2020 (or death); Quality and Outcomes Framework (QOF) read code of type 2 diabetes diagnosis (including codes C10F.00, C10F.11, and complication-related derivatives; full list of included codes given in supplementary file 1).

Exposure

Primary care service utilisation was defined as the total number of contacts during the in-hours service over the study period which was between April 2013 and April 2020. This included telephone, in-person or home visits recorded in the electronic primary care record.

Outcomes

i) Cardiovascular disease (CVD) events, defined as a composite of myocardial infarction, amputation, and stroke
ii) All-cause mortality defined as death from any cause during the study period

Covariates

Baseline sociodemographic data: We extracted data on age, sex and ethnicity. Socioeconomic deprivation was determined by the index of multiple deprivation (IMD), a score calculated by the government to reflect deprivation in a specific geographic area determined based on 7 domains of deprivation including income, employment, education, health, and crime[22].

Baseline clinical variables: Baseline comorbidities were defined from diagnostic codes of existing QOF conditions and included coronary heart disease, chronic kidney disease, chronic obstructive pulmonary disease (COPD), asthma, cancer, dementia, atrial fibrillation, epilepsy, heart failure, stroke, peripheral vascular disease, hypertension, osteoporosis, osteoarthritis, depression and frailty. Frailty was defined using the Electronic Frailty Index Score, a validated tool used to estimate an individual's frailty state using data automatically held in primary care health records [23]. Latest smoking status (current, ex or never-smoked), weight, body mass index (BMI), systolic and diastolic blood pressure and biochemistry measures (including HbA_{1c} total cholesterol, HDL-cholesterol and eGF) were examined as the earliest available recordings from April 1st 2013

Duration of type 2 diabetes: We estimated the interval between the first code for type 2 diabetes in the electronic record until study entry (April 2013).

Statistical Analysis:

Baseline characteristics were summarised using descriptive statistics in Table 1. Time series graphs were plotted using mean utilisation for each quarter over the 7-year follow-up. We compared these between sociodemographic

variables. Service utilisation was summarised with descriptive statistics and divided into tertiles in line with previous approaches in the literature[24] in Table 2; we tested for differences between tertiles using one-way analysis of variance for continuous variables (ANOVA) and a χ^2-test for categorical variables. Finally, we constructed univariate and multivariable logistic models on a complete-case analysis to examine the association between service utilisation (total number of contacts over the study duration), CVD events, and all-cause mortality.

Multivariable logistic regression models were adjusted for potential confounders on *a priori* reasoning including baseline sociodemographic variables (age, sex, ethnicity and IMD); clinical variables to take into account disease severity as sicker patients might attend more frequently (HbA1c, blood pressure, total cholesterol, BMI, frailty, total number of medications prescribed across the study period and smoking status); and practice level co-variates (to account for clustering). Parameter estimates are presented with 95% confidence intervals. Tests were conducted as two-sided, at the 5% significance level. Data were analysed using STATA version 16.1 and R version 4.0.3. Our findings are reported in line with the STROBE and RECORD guidelines for observational studies using routinely collected health data[25].

Ethical Approval

The Hampshire Health Record (Care and Health Information Exchange) has its own independent panel for ethics and governance approval. We have been granted governance and ethics approval. We have additionally received approval from the University of Southampton Research and Governance office (submission reference: ERGO49232).

Patient and Public Involvement

No direct involvement.

Results:

Participant characteristics

The cohort included 110 240 people with type 2 diabetes across 168 primary care practices in Southern England. The mean (SD) age of participants at baseline was 62.9 (14.3) years, there were more men than women (55.8%), and over a quarter of the cohort came from the highest socioeconomic quintile (IMD 5, 26.5%). The characteristics of participants within the cohort are summarised in Table 1. We compared participants with and without missing data, with ethnicity having the largest number of missing data (48.2%), which is not unusual for routinely collected primary care data[26]. The majority of participants with a coded ethnicity were white (90.1%). Participants with missing data were more likely to be male, and from the highest socioeconomic quintile.

Primary care service utilization

Across the whole study period (April 2013 to April 2020), mean number of attendances per patient was 74.0 (2.68 per quarter, SD 2.04). Seasonal variations were observed within each year (with higher use in winter) but overall, mean (annual rolling average) utilisation for the cohort increased over the study period from 2.48 (2.01) per quarter in 2013 to 2.78 (2.06) attendances per quarter in 2020 (Figure 1). This increase was observed across all age groups (Figure 2A) and was highest amongst those aged 65-84 years. Attendance rates were higher in White patients compared to Black, Asian, or other ethnic minority backgrounds; (Figure 2B).

Characteristics of patients by service utilisation tertile

Sociodemographic and clinical characteristics of included individuals are described by service utilisation tertile in Table 2. Mean (SD) utilisation of primary care services was four times greater in the highest utilisation tertile (120 (47.1) attendances) compared to the lowest (38.6(8.9) attendances)(p<0.001). Patients in the highest-usage tertile were more likely to be female, older, more frail, white, from the least deprived IMD quintile, and to have 5 or more

For peer review only - http://bmjopen.bmj.com/site/about/guidelines.xhtml

comorbidities. Patients in the lowest-usage tertile were more likely to be male, younger, non-White, less multimorbid, less frail, and from the most deprived IMD quintile. Mean (SD) HbA1c was higher in the highest utilisation tertile (60.2 (17.65) mmol/mol)) than the lowest utilisation tertile (57.9(16.8) mmol/mol).

Primary care utilisation and outcomes

In both univariate and maximally adjusted models (table 3), higher rates of service utilisation were associated with higher rates of cardiovascular disease (adjusted OR 1.0057 per additional primary care attendance; 95% CI 1.0052 – 1.0061; p<0.001) and mortality (adjusted OR 1.0056; 95% CI 1.0050 – 1.0063; p<0.001).

Discussion

Summary of key findings

In this retrospective cohort study of 110,240 people with type 2 diabetes, we observed growing rates of primary care service utilisation between 2013 and 2020. This was highest amongst White females with multimorbidity and polypharmacy from higher socio-economic backgrounds. After adjusting for potential confounders including age and co-morbidities, we find more frequent contacts with primary care is associated with higher risk of CVD events and mortality. These findings could suggest a need to focus on personalisation of care, and quality of delivered care, to improve outcomes in type 2 diabetes; or could reflect reverse causality, ie, services are being accessed more frequently by those with greater health needs.

Comparison to existing literature

Our findings are in agreement with those of Hobbs et al describing trends of increasing primary care utilisation between 2007 – 2014 in England[19]. While most studies in this domain have examined trends in general utilisation of primary care, our study is novel in specifically examining patters of service use among individuals with type 2 diabetes.

In a recent cross-sectional observational study assessing routine primary care data from 2.7 million English patients, Lay-Flurrie et al report no association between service utilization and mortality[27]. In their analysis, the authors did not stratify by underlying diagnosis, and just 16.5% of included patients were aged over 65 years, in contrast to 50.4% in our cohort. Taken in tandem with our findings that increased service utilisation is associated with mortality in adults with type 2 diabetes, these findings imply service utilisation might offer benefit as a predictor of poor outcomes in selected primary care populations, such as older adults, or those with type 2 diabetes

We find that in patients with type 2 diabetes higher numbers of primary care attendances were associated with a higher odds of cardiovascular events and mortality, even after taking into account co-morbidities and socio-demographic factors. One explanation for this finding could be that optimal preventive care cannot always be achieved in the context of services poor in time and funding resources, despite high frequency contacts. This would be supported by findings from semi-structured interviews in patients with type 2 diabetes, GPs and specialist nurses, which find that only “minimum care standards can be maintained” in providing care to patients with type 2 diabetes[28].

Our study does not inform on the mechanisms underlying the associations between service utilisation, CVD and mortality. It is possible reverse causality[29]could underlie this finding: patients with established cardiovascular disease, for example, are more likely to use primary care services for medication reviews and routine care, and also more likely to develop further cardiovascular disease, or die[30]. Patients who are more frail and multimorbid, or with more difficult to manage or refractory disease, may also be more likely to use primary care services more frequently[31], or to be followed up more intensively by their clinicians, although we adjusted for these potential confounders in our analysis. Alternatively, this finding could reflect underlying mechanisms not captured in our

For peer review only - http://bmjopen.bmj.com/site/about/guidelines.xhtml

adjusted models, such as a higher burden of non-cardiovascular diseases associated with mortality such as cancer and COPD [32].

Strengths and Limitations

Strengths of our study include its large sample of 110,240 people and a relatively long follow-up period of seven years with robust and precise measures of clinical parameters, and restriction of analysis to complete cases. Limitations include the retrospective, observational nature of our study design which limits permissible inferences on causality or directionality of associations; it is unclear from our findings why a relationship between higher rates of service utilisation and poor outcomes exists in type 2 diabetes, and reverse causality may well underlie these findings, ie, more unwell patients requiring more frequent care and being invited for more regular reviews. Our data do not capture information on the nature, quality, or duration of primary care contacts; who the contact was with (eg a GP or specialist nurse); nor the relevance of that contact to type 2 diabetes; for example, patients attending once yearly for a 30 minute review of their type 2 diabetes would appear to be using services to a lesser extent than patients attending three times per year for 10 minute appointments with minor ailments, but may be receiving more focused, high-quality diabetes care. Although the study population is large and includes a large geographic area in the UK, it does include a more affluent, less ethnically diverse and less socioeconomically deprived population than the UK average, which may limit generalisability of findings. Relatively high rates of missing data on participant ethnicity are in keeping with typical results taken from real-world primary care databases, but this missing data is an important limitation to interpretation of findings related to ethnicity, and capture of this data in future observational cohorts would be of clear benefit. Furthermore, we did not further sub-stratify patient groups by, for example, HbA1c levels, or comorbid conditions such as hypertension; this approach could identify particularly high risk groups, and could form the basis for future research in this field.

Implications for Research and Practice

This study shows an increase of 8.3% in rates of primary care service utilization by patients with type 2 diabetes between 2013 and 2020. Highest rates of utilisation were seen in older and frailer patients. High levels of missing data regarding patient ethnicity may limit understanding, and future focus on capturing this within electronic health records may be of benefit in redressing health inequalities.

Our finding that increased service utilisation is not associated with better outcomes in type 2 diabetes suggests simple expansion of number of primary care contacts may not necessarily be beneficial, and that there is a need to consider the quality, nature and content of contacts when tailoring service design in type 2 diabetes. Further research could highlight knowledge gaps in understanding the frequency and content of cardiovascular risk assessments, lifestyle and medication reviews in high risk individuals, for example.

As opposed to achieving better disease control and developing fewer complications, patients with more frequent primary care contacts had higher HbA1c, higher rates of CVD, and higher rates of mortality. Although these patients tended to be older and more multimorbid, these associations persisted after adjustment for these and other confounders. Further research capturing the nature, content and duration of contacts in relation to delivery of care for type 2 diabetes is needed to understand the mechanisms underlying this association and address contributory factors.

Funding statement:

HDM is a National Institute for Health Research (NIHR) funded Academic Clinical Lecturer and has received NIHR School of Primary Care Research funding to support this work (SPCR2014-10043). SH is an Academic Clinical Fellow funded by the NIHR. The views and opinions expressed by authors in this publication are those

For peer review only - http://bmjopen.bmj.com/site/about/guidelines.xhtml

of the authors and do not necessarily reflect those of the UK National Institute for Health Research (NIHR) or the Department of Health and Social Care.

Author contributions:

HDM designed the study, wrote the first draft of the paper, edited and contributed to subsequent versions. SH analysed the data, and drafted and revised subsequent versions of the paper. JH carried out data analysis and revised the paper. HH revised the paper. BS provided advice on statistical methods and revised the paper.

Competing Interests:

None to declare.

Data availability statement

Access to the dataset used for this research is governed by the Hampshire Health Record (Care and Health Information Exchange), which has its own independent panel for ethics and governance approval, and data access. More information on their procedures can be found here: https://careandhealthinformationexchange.org.uk/wp-content/uploads/2018/04/CHIE-Review-of-Procedures-Compliance-with-GDPR_V5-1.pdf.

References:

1 Diabetes UK. Us, diabetes and a lot of facts and stats. *Diabetes UK* 2019;:1–48.www.diabetes.org.uk (accessed 11 Feb 2021).

2 Hex N, Bartlett C, Wright D, *et al.* Estimating the current and future costs of Type1 and Type2 diabetes in the UK, including direct health costs and indirect societal and productivity costs. *Diabet Med* 2012;**29**:855–62. doi:10.1111/j.1464-5491.2012.03698.x

3 National Health Service. The NHS Long Term Plan. 2019. https://www.longtermplan.nhs.uk/publication/nhs-long-term-plan/ (accessed 14 Jan 2021).

4 Davidson JA. The Increasing Role of Primary Care Physicians in Caring for Patients With Type 2 Diabetes Mellitus. *Mayo Clin Proc* 2010;**85**:S3. doi:10.4065/MCP.2010.0466

5 Heald AH, Livingston M, Malipatil N, *et al.* Improving type 2 diabetes mellitus glycaemic outcomes is possible without spending more on medication: Lessons from the UK National Diabetes Audit. *Diabetes, Obes Metab* 2018;**20**:185–94. doi:10.1111/dom.13067

6 Forbes LJL, Marchand C, Doran T, *et al.* The role of the Quality and Outcomes Framework in the care of long-term conditions: A systematic review. *Br J Gen Pract* 2017;**67**:e775–84. doi:10.3399/bjgp17X693077

7 Gadsby R, Young B. Diabetes care in England and Wales: information from the 2010-2011 National Diabetes Audit. *Diabet Med* 2013;**30**:799–802. doi:10.1111/dme.12182

8 Wright CE, Yeung S, Knowles H, *et al.* Factors influencing variation in participation in the National Diabetes Audit and the impact on the Quality and Outcomes Framework indicators of diabetes care management. *BMJ Open Diabetes Res Care* 2018;**6**:e000554. doi:10.1136/bmjdrc-2018-000554

9 International Diabetes Federation. IDF Diabetes Atlas, 7th edn. Brussels, Belgium: International Diabetes Federation 2015. *International Diabetes Federation. IDF Diabetes Atlas, 7th edn. Brussels,*

For peer review only - http://bmjopen.bmj.com/site/about/guidelines.xhtml

Belgium: International Diabetes Federation, 2015.

10 Mc Hugh S, O'Mullane M, Perry IJ, *et al.* Barriers to, and facilitators in, introducing integrated diabetes care in Ireland: a qualitative study of views in general practice. *BMJ Open* 2013;**3**:e003217. doi:10.1136/bmjopen-2013-003217

11 Kutz TL, Roszhart JM, Hale M, *et al.* Improving comprehensive care for patients with diabetes. *BMJ Open Qual* 2018;**7**:e000101. doi:10.1136/bmjoq-2017-000101

12 Bongaerts BWC, Müssig K, Wens J, *et al.* Effectiveness of chronic care models for the management of type 2 diabetes mellitus in Europe: a systematic review and meta-analysis. *BMJ Open* 2017;**7**:e013076. doi:10.1136/BMJOPEN-2016-013076

13 Lim LL, Lau ESH, Kong APS, *et al.* Aspects of Multicomponent Integrated Care Promote Sustained Improvement in Surrogate Clinical Outcomes: A Systematic Review and Meta-analysis. *Diabetes Care* 2018;**41**:1312–20. doi:10.2337/dc17-2010

14 Dambha-Miller H, Cooper AJM, Kinmonth AL, *et al.* Effect on cardiovascular disease risk factors of interventions to alter consultations between practitioners and patients with type 2 diabetes: A systematic review and meta-analysis of trials in primary care. *Heal Expect* Published Online First: February 2017. doi:10.1111/hex.12546

15 Roland M, Everington S. Tackling the crisis in general practice. *BMJ* 2016;**352**:i942. doi:10.1136/BMJ.I942

16 The King's Fund. Public satisfaction with the NHS and social care in 2017. 2017.

17 Maruthappu M, Sood HS, Keogh B. The NHS Five Year Forward View: implications for clinicians. *BMJ* 2014;**349**:g6518.

18 The King's Fund. The NHS long-term plan explained. 2019.

19 Hobbs FDR, Bankhead C, Mukhtar T, *et al.* Clinical workload in UK primary care: a retrospective analysis of 100 million consultations in England, 2007–14. *Lancet* 2016;**387**:2323–30. doi:10.1016/S0140-6736(16)00620-6

20 L.J. H, T.B. D, Renders CM, *et al.* Interventions to improve the management of diabetes mellitus in primary health care and outpatient community settings. L.J. Hansen, Central Forskningsenhed for Almen Praksis og Afdeling for Almen Medicin, Panum Instituttet, Kobenhavns Universitet, DK-2200 Kobenhavn N.: : John Wiley & Sons, Ltd 2002. doi:10.1002/14651858.CD001481

21 Griffin SJ, Kinmonth A-L, Veltman MWM, *et al.* Effect on health-related outcomes of interventions to alter the interaction between patients and practitioners: a systematic review of trials. *Ann Fam Med* 2004;**2**:595–608. doi:10.1370/afm.142

22 Jordan H, Roderick P, Martin D. The Index of Multiple Deprivation 2000 and accessibility effects on health. *J Epidemiol Community Health* 2004;**58**:250–7. doi:10.1136/jech.2003.013011

23 Clegg A, Bates C, Young J, *et al.* Development and validation of an electronic frailty index using routine primary care electronic health record data. *Age Ageing* 2016;**45**:353–60. doi:10.1093/ageing/afw039

24 Turrell G, Oldenburg BF, Harris E, *et al.* Utilisation of general practitioner services by socio-economic disadvantage and geographic remoteness. *Aust N Z J Public Health* 2004;**28**:152–8. doi:10.1111/j.1467-842X.2004.tb00929.x

For peer review only - http://bmjopen.bmj.com/site/about/guidelines.xhtml

25 Benchimol EI, Smeeth L, Guttmann A, *et al.* The REporting of studies Conducted using Observational Routinely-collected health Data (RECORD) Statement. *PLoS Med* 2015;**12**:1001885. doi:10.1371/journal.pmed.1001885

26 Mathur R, Bhaskaran K, Chaturvedi N, *et al.* Completeness and usability of ethnicity data in UK-based primary care and hospital databases. *J Public Heal (United Kingdom)* 2014;**36**:684–92. doi:10.1093/pubmed/fdt116

27 Lay-Flurrie S, Mathieu E, Bankhead C, *et al.* Patient consultation rate and clinical and NHS outcomes: A cross-sectional analysis of English primary care data from 2.7 million patients in 238 practices. *BMC Health Serv Res* 2019;**19**. doi:10.1186/s12913-019-4036-y

28 Dambha-Miller H, Griffin SJ, Kinmonth AL, *et al.* Provision of services in primary care for type 2 diabetes: a qualitative study with patients, GPs, and nurses in the East of England. *Br J Gen Pract* 2020;**70**:E668–75. doi:10.3399/bjgp20X710945

29 Leszczensky L, Wolbring T. How to Deal With Reverse Causality Using Panel Data? Recommendations for Researchers Based on a Simulation Study. *Sociol Methods Res* 2019;:004912411988247. doi:10.1177/0049124119882473

30 Damen JAAG, Hooft L, Schuit E, *et al.* Prediction models for cardiovascular disease risk in the general population: Systematic review. BMJ. 2016;**353**:i2416. doi:10.1136/bmj.i2416

31 Nunes BP, Flores TR, Mielke GI, *et al.* Multimorbidity and mortality in older adults: A systematic review and meta-analysis. Arch. Gerontol. Geriatr. 2016;**67**:130–8. doi:10.1016/j.archger.2016.07.008

32 Burney P, Jithoo A, Kato B, *et al.* Chronic obstructive pulmonary disease mortality and prevalence: The associations with smoking and poverty-A bold analysis. *Thorax* 2014;**69**:465–73. doi:10.1136/thoraxjnl-2013-204460

For peer review only

For peer review only - http://bmjopen.bmj.com/site/about/guidelines.xhtml

For peer review only

Table 1: Summary sociodemographic and clinical characteristics of the Care and Health Information Analytics type 2 diabetes cohort, comprising all patients diagnosed with type 2 diabetes in a linked primary and secondary care electronic database in Hampshire, UK (n = 110,240).

Sex		Patients Missing Data n (%)
Female n(%)	48706 (44.18)	0 (0)
Male n(%)	61534 (55.82)	
Age (years)		
Age	62.89 (14.29)	0 (0)
Ethnicity		
Asian n(%)	3295 (2.99)	54,011 (48.99)
Black n(%)	498 (0.45)	
Mixed or Other n(%)	810 (0.73)	
White n(%)	51626 (46.83)	
Index of Multiple Deprivation		
1 n(%)	14035 (12.73)	1075 (0.98)
2 n(%)	21833 (19.8)	
3 n(%)	21064 (19.11)	
4 n(%)	23062 (20.92)	
5 n(%)	29171 (26.46)	
Smoking Status		
Current Smoker n(%)	13875 (12.59)	492 (0.45)
Ex Smoker n(%)	44875 (40.71)	
Never Smoked n(%)	50998 (46.26)	
Total co-morbidities		
Total co-morbidities	2.36 (1.79)	0 (0)

For peer review only - http://bmjopen.bmj.com/site/about/guidelines.xhtml

Individual co-morbidities		
Stroke n(%)	7694 (6.98)	0 (0)
Myocardial Infarction n(%)	9812 (8.9)	0 (0)
Peripheral Vascular Disease n(%)	3565 (3.23)	0 (0)
Chronic Kidney Disease n(%)	15471 (14.03)	0 (0)
Dementia n(%)	3558 (3.23)	0 (0)
Total Medications Prescribed in study period (	48.59 (30.12)	0 (0)
Frailty Score	0.22 (0.11)	77 (0.07)
Values are presented as mean (standard deviation) unless otherwise stated, all values are taken at baseline. HbA_{1c} = glycated haemoglobin		

Table 2: Sociodemographic and clinical characteristics of the Care and Health Information Analytics type 2 diabetes cohort, presented by primary care utilisation tertiles, comprising all patients diagnosed with type 2 diabetes in a linked primary and secondary care electronic database in Hampshire, UK. (n = 75,701).

**Variables**	**All Participants**	**Tertile 1 (low usage)**	**Tertile 2**	**Tertile 3 (high usage)**	**p-value**
Complete case cohort					
Number of patients (n)	75701 (0)	25234 (0)	25234 (0)	25233 (0)	
Average attendances mean (SD)	74.04 (44)	38.6 (8.49)	63.8 (7.87)	119.73 (47.17)	<0.01
Sex					
Female n(%)	34887 (46.09)	9959 (39.47)	11739 (46.52)	13189 (52.27)	<0.01
Male n(%)	40814 (53.91)	15275 (60.53)	13495 (53.48)	12044 (47.73)	<0.01
Age at start (years)					
Age mean(SD)	63.15 (13.04)	60.2 (12.78)	63.05 (12.77)	66.19 (12.87)	<0.01
Age group at start (years)					
18-44 years n(%)	6506 (8.59)	2770 (10.98)	2102 (8.33)	1634 (6.48)	<0.01
45-64 years n(%)	31054 (41.02)	12436 (49.28)	10491 (41.57)	8127 (32.21)	<0.01

For peer review only - http://bmjopen.bmj.com/site/about/guidelines.xhtml

65-84 years n(%)	35804 (47.3)	9510 (37.69)	11931 (47.28)	14363 (56.92)	<0.01
85+ years n(%)	2337 (3.09)	518 (2.05)	710 (2.81)	1109 (4.4)	<0.01
Ethnicity					
Asian n(%)	2097 (2.77)	730 (2.89)	841 (3.33)	526 (2.08)	<0.01
Black n(%)	309 (0.41)	110 (0.44)	118 (0.47)	81 (0.32)	0.03
Mixed or Other n(%)	544 (0.72)	195 (0.77)	182 (0.72)	167 (0.66)	0.34
White n(%)	39519 (52.2)	11272 (44.67)	13543 (53.67)	14704 (58.27)	<0.01
Missing Data n(%)	33232 (43.9)	12927 (51.23)	10550 (41.81)	9755 (38.66)	<0.01
Index of Multiple Deprivation Quintile					
1 n(%)	9301 (12.29)	3311 (13.12)	3013 (11.94)	2977 (11.8)	<0.01
2 n(%)	14215 (18.78)	4857 (19.25)	4621 (18.31)	4737 (18.77)	0.01
3 n(%)	13924 (18.39)	4889 (19.37)	4477 (17.74)	4558 (18.06)	0.56
4 n(%)	16285 (21.51)	5415 (21.46)	5560 (22.03)	5310 (21.04)	0.01
5 n(%)	21368 (28.23)	6612 (26.2)	7348 (29.12)	7408 (29.36)	0.03
Missing IMD Values n(%)	608 (0.8)	150 (0.59)	215 (0.85)	243 (0.96)	<0.01
HbA1c (%)					
HbA1c mean(SD)	7.55 (1.53)	7.43 (1.48)	7.51 (1.49)	7.65 (1.59)	<0.01
HbA1c (mmol/mol)					
HbA1c mean(SD)	59.1 (16.97)	57.85 (16.38)	58.73 (16.49)	60.21 (17.65)	<0.01
BMI (kg)/m^2					
BMI mean(SD)	31.9 (7.31)	31.2 (7.21)	31.1 (7.19)	32.2 (7.38)	<0.01
Electronic frailty index score					
Mean Electronic frailty index score (SD)	0.22 (0.10)	0.16 (0.08)	0.22 (0.09)	0.30 (0.11)	<0.001
Number of co-morbidities					
<= 2 n(%)	40400 (53.37)	18048 (71.52)	13659 (54.13)	8693 (34.45)	<0.01
3-4 n(%)	23970 (31.66)	5996 (23.76)	8575 (33.98)	9399 (37.25)	<0.01
>=5 n(%)	11331 (14.97)	1190 (4.72)	3000 (11.89)	7141 (28.3)	<0.01
Total number of medications prescribed					
<=2 n(%)	1078 (1.42)	737 (2.92)	235 (0.93)	106 (0.42)	<0.01

For peer review only - http://bmjopen.bmj.com/site/about/guidelines.xhtml

3-5 n(%)	1037 (1.37)	674 (2.67)	285 (1.13)	78 (0.31)	<0.01
6-9 n(%)	1498 (1.98)	893 (3.54)	419 (1.66)	186 (0.74)	<0.01
>=10 n(%)	72088 (95.23)	22930 (90.87)	24295 (96.28)	24863 (98.53)	<0.01

Data sourced from CHIA dataset Values are presented as mean (standard deviation) unless otherwise stated, all values are taken at baseline. HbA_{1c} = glycated haemoglobin)

For peer review only

Table 3: Univariate (unadjusted) and multivariate (adjusted) models describing the association between primary care service utilisation, defined as number of contacts with primary care services, cardiovascular disease and mortality, in the CHIA data set between 2013 and 2020

	**Unadjusted odds ratio (95% CI) per additional primary care contact**	**Univariate P Value**	**Adjusted** odds ratio (95% CI) per additional primary care contact**	**Multivariate P value**
**Cardiovascular disease***	1.0071 (1.0066 - 1.0074)	<0.001	1.0057 (1.0052 - 1.0061)	<0.001
**Mortality**	1.0077 (1.0070 - 1.0082)	<0.001	1.0056 (1.0050 - 1.0063)	<0.001

** Adjusted for age, sex, ethnicity, index of multiple deprivation, smoking status, number of prescribed medications, frailty, BMI, HbA1c, systolic and diastolic blood pressure, mean total cholesterol, and practice-level co-morbidity prevalence to account for local clustering.

* defined as a composite of myocardial infarction, amputation, and stroke

For peer review only - http://bmjopen.bmj.com/site/about/guidelines.xhtml

Figure 1: Mean number of primary care attendances by year quarter (Q1-Q4) between 2013 and 2020 in the CHIA type 2 diabetes cohort. Q1 is defined as January – March.

For peer review only

For peer review only - http://bmjopen.bmj.com/site/about/guidelines.xhtml

Figure 2: Rolling four-quarter mean number of primary care attendances by year quarter between 2013 and 2020 in the CHIA type 2 diabetes cohort stratified by (A) age band (in years) at study entry and (B) ethnicity. Q1 is defined as January – March. For example, the mean number of attendances for Q1 2014 represents mean attendance between Q1 2013 and Q1 2014.

For peer review only

For peer review only - http://bmjopen.bmj.com/site/about/guidelines.xhtml

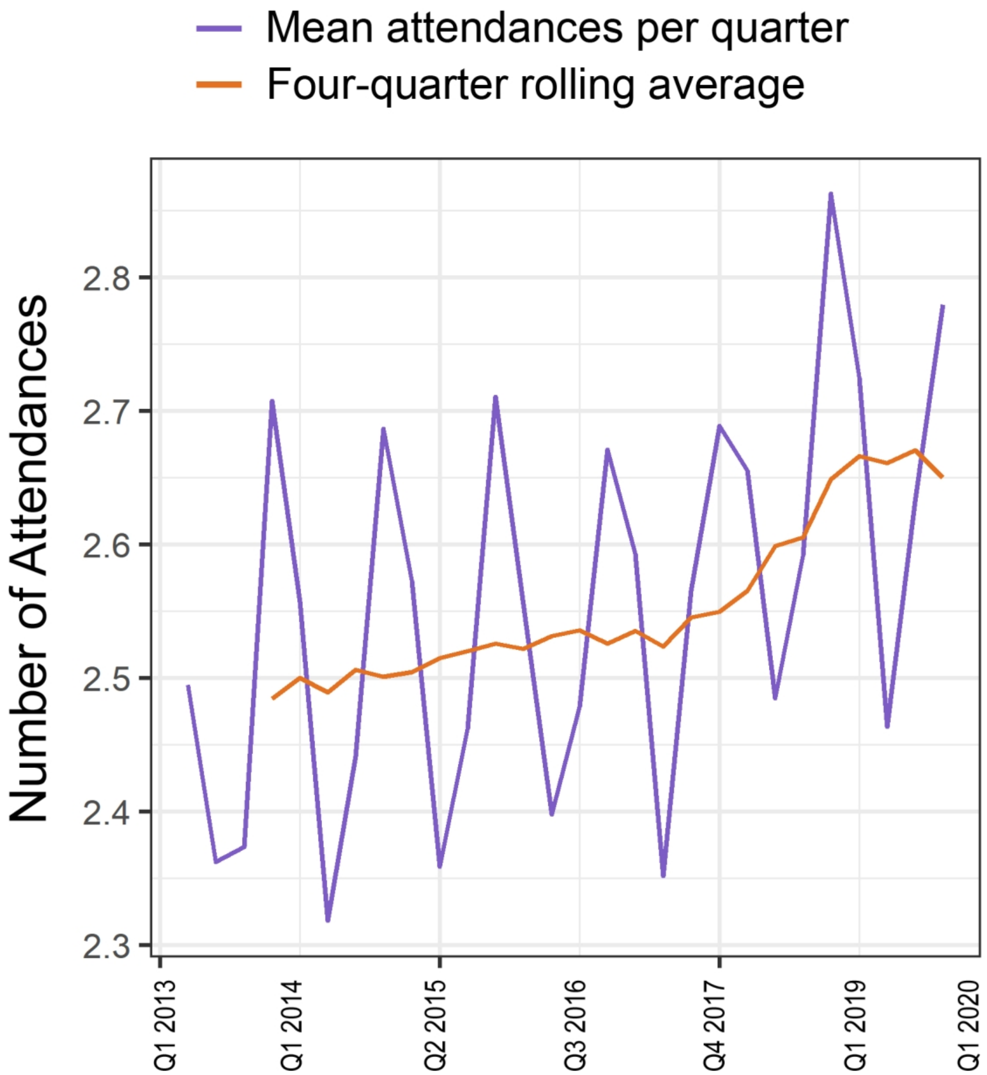

Figure 1: Mean number of primary care attendances by year quarter (Q1-Q4) between 2013 and 2020 in the CHIA type 2 diabetes cohort. Q1 is defined as January – March.

Supplementary File 1: Table to show diagnostic read codes used to identify individuals with type 2 diabetes to include in analysis

Read Code	Condition
C10F.00	Type 2 diabetes mellitus
C10F000	Type 2 diabetes mellitus with renal complications
C10F011	Type II diabetes mellitus with renal complications
C10F100	Type 2 diabetes mellitus with ophthalmic complications
C10F.11	Type II diabetes mellitus
C10F111	Type II diabetes mellitus with ophthalmic complications
C10F200	Type 2 diabetes mellitus with neurological complications
C10F211	Type II diabetes mellitus with neurological complications
C10F300	Type 2 diabetes mellitus with multiple complications
C10F311	Type II diabetes mellitus with multiple complications
C10F400	Type 2 diabetes mellitus with ulcer
C10F411	Type II diabetes mellitus with ulcer
C10F500	Type 2 diabetes mellitus with gangrene
C10F511	Type II diabetes mellitus with gangrene
C10F600	Type 2 diabetes mellitus with retinopathy
C10F611	Type II diabetes mellitus with retinopathy
C10F700	Type 2 diabetes mellitus - poor control
C10F711	Type II diabetes mellitus - poor control
C10F800	Reaven's syndrome
C10F811	Metabolic syndrome X
C10F900	Type 2 diabetes mellitus without complication

For peer review only

C10F911	Type II diabetes mellitus without complication
C10FA00	Type 2 diabetes mellitus with mononeuropathy
C10FA11	Type II diabetes mellitus with mononeuropathy
C10FB00	Type 2 diabetes mellitus with polyneuropathy
C10FB11	Type II diabetes mellitus with polyneuropathy
C10FC00	Type 2 diabetes mellitus with nephropathy
C10FC11	Type II diabetes mellitus with nephropathy
C10FD00	Type 2 diabetes mellitus with hypoglycaemic coma
C10FD11	Type II diabetes mellitus with hypoglycaemic coma
C10FE00	Type 2 diabetes mellitus with diabetic cataract
C10FE11	Type II diabetes mellitus with diabetic cataract
C10FF00	Type 2 diabetes mellitus with peripheral angiopathy
C10FG00	Type 2 diabetes mellitus with arthropathy
C10FG11	Type II diabetes mellitus with arthropathy
C10FH00	Type 2 diabetes mellitus with neuropathic arthropathy
C10FJ00	Insulin treated Type 2 diabetes mellitus
C10FJ11	Insulin treated Type II diabetes mellitus
C10FK00	Hyperosmolar non-ketotic state in type 2 diabetes mellitus
C10FL00	Type 2 diabetes mellitus with persistent proteinuria
C10FL11	Type II diabetes mellitus with persistent proteinuria
C10FM00	Type 2 diabetes mellitus with persistent microalbuminuria
C10FM11	Type II diabetes mellitus with persistent microalbuminuria
C10FN00	Type 2 diabetes mellitus with ketoacidosis

For peer review only

C10FP00	Type 2 diabetes mellitus with ketoacidotic coma
C10FQ00	Type 2 diabetes mellitus with exudative maculopathy
C10FR00	Type 2 diabetes mellitus with gastroparesis

For peer review only

BMJ Open

Primary care service utilisation and outcomes in type 2 diabetes: A longitudinal cohort analysis

Journal:	*BMJ Open*
Manuscript ID	bmjopen-2021-054654.R2
Article Type:	Original research
Date Submitted by the Author:	04-Jan-2022
Complete List of Authors:	Hodgson, Sam; University of Southampton, Primary Care Research Centre<br>Morgan-Harrisskitt, Jeffrey; NHS South Central and West Commissioning Support Unit<br>Hounkpatin, Hilda; University of Southampton, Primary Care Research Centre<br>Stuart, Beth; University of Southampton<br>Dambha-Miller, Hajira; University of Southampton, Primary Care Research Centre
**Primary Subject Heading**:	Diabetes and endocrinology
Secondary Subject Heading:	General practice / Family practice
Keywords:	PRIMARY CARE, General diabetes < DIABETES & ENDOCRINOLOGY, Organisation of health services < HEALTH SERVICES ADMINISTRATION & MANAGEMENT

SCHOLARONE™
Manuscripts

I, the Submitting Author has the right to grant and does grant on behalf of all authors of the Work (as defined in the below author licence), an exclusive licence and/or a non-exclusive licence for contributions from authors who are: i) UK Crown employees; ii) where BMJ has agreed a CC-BY licence shall apply, and/or iii) in accordance with the terms applicable for US Federal Government officers or employees acting as part of their official duties; on a worldwide, perpetual, irrevocable, royalty-free basis to BMJ Publishing Group Ltd ("BMJ") its licensees and where the relevant Journal is co-owned by BMJ to the co-owners of the Journal, to publish the Work in this journal and any other BMJ products and to exploit all rights, as set out in our licence.

The Submitting Author accepts and understands that any supply made under these terms is made by BMJ to the Submitting Author unless you are acting as an employee on behalf of your employer or a postgraduate student of an affiliated institution which is paying any applicable article publishing charge ("APC") for Open Access articles. Where the Submitting Author wishes to make the Work available on an Open Access basis (and intends to pay the relevant APC), the terms of reuse of such Open Access shall be governed by a Creative Commons licence – details of these licences and which Creative Commons licence will apply to this Work are set out in our licence referred to above.

Other than as permitted in any relevant BMJ Author's Self Archiving Policies, I confirm this Work has not been accepted for publication elsewhere, is not being considered for publication elsewhere and does not duplicate material already published. I confirm all authors consent to publication of this Work and authorise the granting of this licence.

For peer review only

Primary care service utilisation and outcomes in type 2 diabetes: A longitudinal cohort analysis

Sam Hodgson[1], Jeffrey Morgan-Harrisskitt[2], Hilda Hounkpatin[1], Beth Stuart[1] and Hajira Dambha-Miller[1]

1. Primary Care Research Centre, University of Southampton, UK
2. Strategy and Transformation Department, NHS South, Central and West, UK

Correspondence: Dr Sam Hodgson, Primary Care Research Centre, University of Southampton, Southampton, SO16 5ST, Email: S.c.b.hodgson@soton.ac.uk / samcbhodgson@gmail.com

Keywords: type 2 diabetes; primary care; service utilisation; stratification

Word count

Main text: 2505

Abstract: 289/300

For peer review only

For peer review only - http://bmjopen.bmj.com/site/about/guidelines.xhtml

Abstract

Objectives: To describe primary care utilisation patterns amongst adults with type 2 diabetes and to quantify the association between utilisation and long-term health outcomes.

Design: Retrospective cohort study

Setting: 168 primary care practices in Southern England within the Electronic Care and Health Information Analytics (CHIA) database between 2013 and 2020

Participants: 110,240 adults with Quality and Outcomes Framework read code of type 2 diabetes diagnosis; age greater than 18 years; linked and continuous records available from April 2013 until April 2020 (or death).

Primary and secondary outcome measures: 1) rates of service utilisation (total number of primary care contacts per quarter) across the study period; 2) participant characteristics associated with higher and lower rates of service utilisation; 3) associations between service utilisation and: i) cardiovascular disease (CVD events), and ii) all-cause mortality.

Results: Mean (standard deviation) number of primary care attendances per quarter in the cohort of 110,240 went from 2.49 (2.01) in 2013 to 2.78 (2.06) in 2020. Patients in the highest-usage tertile were more likely to be female, older, more frail, white, from the least deprived quintile, and to have 5 or more comorbidities. In adjusted models, higher rates of service utilisation (per consultation) were associated with higher rates of CVD events (odds ratio (OR) 1.0058; 95% CI 1.0053 – 1.0062; $p<0.001$) and mortality (OR 1.0057; 95% CI 1.0051 – 1.0064; $p<0.001$)

Conclusions: People with type 2 diabetes are using primary care services more frequently, but increased volume of clinical care does not correlate with better outcomes, although this finding may be driven by more unwell patients contacting services more frequently. Further research on the nature and content of contacts is required to understand how to tailor services to deliver effective care to those at greatest risk of complications.

Article Summary / Strengths and limitations of this study

- This study includes a large sample of 110,240 individuals with a relatively long follow-up period of seven years.
- This study analyses robust and precise measures of clinical parameters from real-world clinical data.
- The retrospective and observational nature of the study design limits permissible inferences on causality or directionality of associations.
- Our data do not capture information on the nature, quality, or duration of primary care contacts, nor the relevance of that contact to type 2 diabetes.
- Although the study population is large, it is taken from an area of the UK (Southern England) which is more affluent, less ethnically diverese, and less socioeconomically deprived than the UK average, which may limit generalisability of findings.

For peer review only - http://bmjopen.bmj.com/site/about/guidelines.xhtml

Introduction:

Diabetes UK estimate that by 2030 5.5 million individuals in the United Kingdom (UK) will be diagnosed with type 2 diabetes,[1] which today costs the National Health Service (NHS) over £10 billion a year.[2] Strategies to optimise treatment and prevent associated complications are therefore a public health priority. The UK Government's NHS long-term plan builds on the General Practice Forward View articulating the need for better prevention of cardiovascular disease (CVD).[3] One key area of focus is the prevention of type 2 diabetes and its related CVD complications in primary care, where over 80% of the disease is managed.[4] It is hypothesised that primary care contacts could provide opportunities to positively influence and support healthy behaviours, optimise treatment plans, promote self-management and detect ill-health earlier.

For this reason, the last few years have seen greater provision of primary care diabetes services through increased multidisciplinary teams, automated recall and greater efforts at monitoring of diabetes progress through the UK National Diabetes Audit,[5] a national surveillance programme assessing the quality of type 2 diabetes care in UK primary care, and Quality Outcome Framework (QOF).[6] However, both the QOF and National Diabetes Audit have demonstrated large variations in primary care diabetes uptake while long-term outcomes remain poor.[7,8] Type 2 diabetes is still the leading cause of premature mortality and for someone diagnosed at the age of 50 years, for example, average life expectancy in the UK is reduced by six years.[9] Despite increased research, funding and the development of a number of interventions to prevent diabetes complications, few have been effectively translated in primary care. [10–13] In part, this may reflect the fact that national strategies and previous diabetes research do not adequately consider the contextual or real-world challenges in primary care. To develop effective strategies that are relevant and applicable to this setting, it is necessary to first understand the current utilisation and availability of primary care services to understand the context in which any future interventions would be nested. [14]

These contextual issues are critical to examine at a time when primary care is rapidly adapting to increasing demands on services, a growing burden of chronic disease and challenges in workforce recruitment. Cuts to public health and social funding with no planned increases in capital, education, training and local authority public health or social care are likely to add to existing pressures. [15–18] Several studies have already described patterns of service utilisation, characteristics of service users, and how these relate to health outcomes in general terms, but these have not specifically considered type 2 diabetes in primary care,[19] nor long-term complications such as heart attacks and strokes that have the greatest impact of patient's lives and on healthcare costs. [20,21]

The availability of longitudinal electronic primary care health records that include large cohorts with objective measures provides an opportunity to overcome the limitations of previous research in this area and inform future provision. In this study, we examined patterns of primary care service utilisation in adults with type 2 diabetes in a large population-based cohort, and quantified the association between utilisation and long-term health outcomes.

Method:

Design

Retrospective cohort analysis

Data source

The Electronic Care and Health Information Analytics (CHIA) database includes individual level anonymised live data from primary care records linked to local acute hospital trusts in the South of England (Hampshire). Data from

For peer review only - http://bmjopen.bmj.com/site/about/guidelines.xhtml

1.5 million consenting patient medical records have been anonymised and collected continuously across 168 primary care practices covering urban and rural populations. Patients automatically “opt in” to their anonymised data being included in the research data set, and can opt out via their primary care provider or the CHIE platform. Data on the proportion of patients opting out was not available to the research team. Data includes coded clinical entries on demographic data, service utilisation, diagnoses, investigations, medications and clinical outcomes from primary care and hospital laboratories.

Study population

Individuals were included if they met the following criteria: age greater than 18 years; linked and continuous records available from April 2013 until April 2020 (or death); Quality and Outcomes Framework (QOF) read code of type 2 diabetes diagnosis (including codes C10F.00, C10F.11, and complication-related derivatives; full list of included codes given in supplementary file 1).

Exposure

Primary care service utilisation was defined as the total number of contacts during the in-hours service over the study period which was between April 2013 and April 2020. This included telephone, in-person or home visits recorded in the electronic primary care record.

Outcomes

i) Cardiovascular disease (CVD) events, defined as a composite of myocardial infarction, amputation, and stroke
ii) All-cause mortality defined as death from any cause during the study period

Covariates

Baseline sociodemographic data: We extracted data on age, sex and ethnicity. Socioeconomic deprivation was determined by the index of multiple deprivation (IMD), a score calculated by the government to reflect deprivation in a specific geographic area determined based on 7 domains of deprivation including income, employment, education, health, and crime[22].

Baseline clinical variables: Baseline comorbidities were defined from diagnostic codes of existing QOF conditions and included coronary heart disease, chronic kidney disease, chronic obstructive pulmonary disease (COPD), asthma, cancer, dementia, atrial fibrillation, epilepsy, heart failure, stroke, peripheral vascular disease, hypertension, osteoporosis, osteoarthritis, depression and frailty. Frailty was defined using the Electronic Frailty Index Score, a validated tool used to estimate an individual’s frailty state using data automatically held in primary care health records [23]. Latest smoking status (current, ex or never-smoked), weight, body mass index (BMI), systolic and diastolic blood pressure and biochemistry measures (including HbA_{1c} total cholesterol, HDL-cholesterol and eGF) were examined as the earliest available recordings from April 1st 2013

Duration of type 2 diabetes: We estimated the interval between the first code for type 2 diabetes in the electronic record until study entry (April 2013).

Statistical Analysis:

Baseline characteristics were summarised using descriptive statistics in Table 1. Time series graphs were plotted using mean utilisation for each quarter over the 7-year follow-up. We compared these between sociodemographic

variables. Service utilisation was summarised with descriptive statistics and divided into tertiles in line with previous approaches in the literature[24] in Table 2; we tested for differences between tertiles using one-way analysis of variance for continuous variables (ANOVA) and a χ^2-test for categorical variables. Finally, we constructed univariate and multivariable logistic models on a complete-case analysis to examine the association between service utilisation (total number of contacts over the study duration), CVD events, and all-cause mortality.

Multivariable logistic regression models were adjusted for potential confounders on *a priori* reasoning including baseline sociodemographic variables (age, sex, ethnicity and IMD); clinical variables to take into account disease severity as sicker patients might attend more frequently (HbA1c, blood pressure, total cholesterol, BMI, frailty, total number of medications prescribed across the study period and smoking status); and practice level co-variates (to account for clustering). Parameter estimates are presented with 95% confidence intervals. Tests were conducted as two-sided, at the 5% significance level. Data were analysed using STATA version 16.1 and R version 4.0.3. Our findings are reported in line with the STROBE and RECORD guidelines for observational studies using routinely collected health data[25].

Ethical Approval

The Hampshire Health Record (Care and Health Information Exchange) has its own independent panel for ethics and governance approval. We have been granted governance and ethics approval. We have additionally received approval from the University of Southampton Research and Governance office (submission reference: ERGO49232).

Patient and Public Involvement

Patients and the public were not directly involved in this research. The nature of the anonymised records meang individual participants could not be involved.

Results:

Participant characteristics

The cohort included 110 240 people with type 2 diabetes across 168 primary care practices in Southern England. The mean (SD) age of participants at baseline was 62.9 (14.3) years, there were more men than women (55.8%), and over a quarter of the cohort came from the highest socioeconomic quintile (IMD 5, 26.5%). The characteristics of participants within the cohort are summarised in Table 1. We compared participants with and without missing data, with ethnicity having the largest number of missing data (48.2%), which is not unusual for routinely collected primary care data[26]. The majority of participants with a coded ethnicity were white (90.1%). Participants with missing data were more likely to be male, and from the highest socioeconomic quintile.

Primary care service utilization

Across the whole study period (April 2013 to April 2020), mean number of attendances per patient was 74.0 (2.68 per quarter, SD 2.04). Seasonal variations were observed within each year (with higher use in winter) but overall, mean (annual rolling average) utilisation for the cohort increased over the study period from 2.48 (2.01) per quarter in 2013 to 2.78 (2.06) attendances per quarter in 2020 (Figure 1). This increase was observed across all age groups (Figure 2A) and was highest amongst those aged 65-84 years. Attendance rates were higher in White patients compared to Black, Asian, or other ethnic minority backgrounds; (Figure 2B).

Characteristics of patients by service utilisation tertile

Sociodemographic and clinical characteristics of included individuals are described by service utilisation tertile in Table 2. Mean (SD) utilisation of primary care services was four times greater in the highest utilisation tertile (120 (47.1) attendances) compared to the lowest (38.6(8.9) attendances)($p<0.001$). Patients in the highest-usage tertile

For peer review only - http://bmjopen.bmj.com/site/about/guidelines.xhtml

were more likely to be female, older, more frail, white, from the least deprived IMD quintile, and to have 5 or more comorbidities. Patients in the lowest-usage tertile were more likely to be male, younger, non-White, less multimorbid, less frail, and from the most deprived IMD quintile. Mean (SD) HbA1c was higher in the highest utilisation tertile (60.2 (17.65) mmol/mol)) than the lowest utilisation tertile (57.9(16.8) mmol/mol).

Primary care utilisation and outcomes

In both univariate and maximally adjusted models (table 3), higher rates of service utilisation were associated with higher rates of CVD (adjusted OR 1.0057 per additional primary care attendance; 95% CI 1.0052 – 1.0061; p<0.001) and mortality (adjusted OR 1.0056; 95% CI 1.0050 – 1.0063; p<0.001).

Discussion

Summary of key findings

In this retrospective cohort study of 110,240 people with type 2 diabetes, we observed growing rates of primary care service utilisation between 2013 and 2020. This was highest amongst White females with multimorbidity and polypharmacy from higher socio-economic backgrounds. After adjusting for potential confounders including age and co-morbidities, we find more frequent contacts with primary care is associated with higher risk of CVD events and mortality. These findings could suggest a need to focus on personalisation of care, and quality of delivered care, to improve outcomes in type 2 diabetes; or could reflect reverse causality, ie, services are being accessed more frequently by those with greater health needs.

Comparison to existing literature

Our findings are in agreement with those of Hobbs et al describing trends of increasing primary care utilisation between 2007 – 2014 in England[19]. While most studies in this domain have examined trends in general utilisation of primary care, our study is novel in specifically examining patters of service use among individuals with type 2 diabetes.

In a recent cross-sectional observational study assessing routine primary care data from 2.7 million English patients, Lay-Flurrie et al report no association between service utilization and mortality[27]. In their analysis, the authors did not stratify by underlying diagnosis, and just 16.5% of included patients were aged over 65 years, in contrast to 50.4% in our cohort. Taken in tandem with our findings that increased service utilisation is associated with mortality in adults with type 2 diabetes, these findings imply service utilisation might offer benefit as a predictor of poor outcomes in selected primary care populations, such as older adults, or those with type 2 diabetes

We find that in patients with type 2 diabetes higher numbers of primary care attendances were associated with a higher odds of CVD and mortality, even after taking into account co-morbidities and socio-demographic factors. One explanation for this finding could be that optimal preventive care cannot always be achieved in the context of services poor in time and funding resources, despite high frequency contacts. This would be supported by findings from semi-structured interviews in patients with type 2 diabetes, GPs and specialist nurses, which find that only "minimum care standards can be maintained" in providing care to patients with type 2 diabetes[28].

Our study does not inform on the mechanisms underlying the associations between service utilisation, CVD and mortality. It is possible reverse causality[29]could underlie this finding: patients with established CVD, for example, are more likely to use primary care services for medication reviews and routine care, and also more likely to develop further CVD, or die[30]. Patients who are more frail and multimorbid, or with more difficult to manage or refractory disease, may also be more likely to use primary care services more frequently[31], or to be followed up more intensively by their clinicians, although we adjusted for these potential confounders in our analysis. Alternatively, this finding could reflect underlying mechanisms not captured in our adjusted models, such as a higher burden of non-cardiovascular diseases associated with mortality such as cancer and COPD [32].

For peer review only - http://bmjopen.bmj.com/site/about/guidelines.xhtml

Strengths and Limitations

Strengths of our study include its large sample of 110,240 people and a relatively long follow-up period of seven years with robust and precise measures of clinical parameters, and restriction of analysis to complete cases. Limitations include the retrospective, observational nature of our study design which limits permissible inferences on causality or directionality of associations; it is unclear from our findings why a relationship between higher rates of service utilisation and poor outcomes exists in type 2 diabetes, and reverse causality may well underlie these findings, ie, more unwell patients requiring more frequent care and being invited for more regular reviews. Our data do not capture information on the nature, quality, or duration of primary care contacts; who the contact was with (eg a GP or specialist nurse); nor the relevance of that contact to type 2 diabetes. For example, patients attending once yearly for a 30 minute review of their type 2 diabetes would appear to be using services to a lesser extent than patients attending three times per year for 10 minute appointments with minor ailments, but may be receiving more focused, high-quality diabetes care. Although the study population is large and includes a large geographic area in the UK, it does include a more affluent, less ethnically diverse and less socioeconomically deprived population than the UK average, which may limit generalisability of findings. Relatively high rates of missing data on participant ethnicity are in keeping with typical results taken from real-world primary care databases, but this missing data is an important limitation to interpretation of findings related to ethnicity, and capture of this data in future observational cohorts would be of clear benefit. Furthermore, we did not further sub-stratify patient groups by, for example, HbA1c levels, or comorbid conditions such as hypertension.Tthis approach could identify particularly high risk groups, and could form the basis for future research in this field.

Implications for Research and Practice

This study shows an increase of 8.3% in rates of primary care service utilization by patients with type 2 diabetes between 2013 and 2020. Highest rates of utilisation were seen in older and frailer patients. High levels of missing data regarding patient ethnicity may limit understanding, and future focus on capturing this within electronic health records may be of benefit in redressing health inequalities.

Our finding that increased service utilisation is not associated with better outcomes in type 2 diabetes suggests simple expansion of number of primary care contacts may not necessarily be beneficial, and that there is a need to consider the quality, nature and content of contacts when tailoring service design in type 2 diabetes. Further research could highlight knowledge gaps in understanding the frequency and content of cardiovascular risk assessments, lifestyle and medication reviews in high risk individuals, for example.

As opposed to achieving better disease control and developing fewer complications, patients with more frequent primary care contacts had higher HbA1c, higher rates of CVD, and higher rates of mortality. Although these patients tended to be older and more multimorbid, these associations persisted after adjustment for these and other confounders. Further research capturing the nature, content and duration of contacts in relation to delivery of care for type 2 diabetes is needed to understand the mechanisms underlying this association and address contributory factors.

Funding statement:

HDM is a National Institute for Health Research (NIHR) funded Academic Clinical Lecturer and has received NIHR School of Primary Care Research funding to support this work (SPCR2014-10043). SH is an Academic Clinical Fellow funded by the NIHR. The views and opinions expressed by authors in this publication are those of the authors and do not necessarily reflect those of the UK National Institute for Health Research (NIHR) or the Department of Health and Social Care.

For peer review only - http://bmjopen.bmj.com/site/about/guidelines.xhtml

Author contributions:

HDM designed the study, wrote the first draft of the paper, edited and contributed to subsequent versions. SH analysed the data, and drafted and revised subsequent versions of the paper. JH carried out data analysis and revised the paper. HH revised the paper. BS provided advice on statistical methods and revised the paper.

Competing Interests:

None to declare.

Data availability statement

Access to the dataset used for this research is governed by the Hampshire Health Record (Care and Health Information Exchange), which has its own independent panel for ethics and governance approval, and data access. More information on their procedures can be found here: https://careandhealthinformationexchange.org.uk/wp-content/uploads/2018/04/CHIE-Review-of-Procedures-Compliance-with-GDPR_V5-1.pdf.

References:

1 Diabetes UK. Us, diabetes and a lot of facts and stats. *Diabetes UK* 2019;:1–48.www.diabetes.org.uk (accessed 11 Feb 2021).

2 Hex N, Bartlett C, Wright D, *et al.* Estimating the current and future costs of Type1 and Type2 diabetes in the UK, including direct health costs and indirect societal and productivity costs. *Diabet Med* 2012;**29**:855–62. doi:10.1111/j.1464-5491.2012.03698.x

3 National Health Service. The NHS Long Term Plan. 2019. https://www.longtermplan.nhs.uk/publication/nhs-long-term-plan/ (accessed 14 Jan 2021).

4 Davidson JA. The Increasing Role of Primary Care Physicians in Caring for Patients With Type 2 Diabetes Mellitus. *Mayo Clin Proc* 2010;**85**:S3. doi:10.4065/MCP.2010.0466

5 Heald AH, Livingston M, Malipatil N, *et al.* Improving type 2 diabetes mellitus glycaemic outcomes is possible without spending more on medication: Lessons from the UK National Diabetes Audit. *Diabetes, Obes Metab* 2018;**20**:185–94. doi:10.1111/dom.13067

6 Forbes LJL, Marchand C, Doran T, *et al.* The role of the Quality and Outcomes Framework in the care of long-term conditions: A systematic review. *Br J Gen Pract* 2017;**67**:e775–84. doi:10.3399/bjgp17X693077

7 Gadsby R, Young B. Diabetes care in England and Wales: information from the 2010-2011 National Diabetes Audit. *Diabet Med* 2013;**30**:799–802. doi:10.1111/dme.12182

8 Wright CE, Yeung S, Knowles H, *et al.* Factors influencing variation in participation in the National Diabetes Audit and the impact on the Quality and Outcomes Framework indicators of diabetes care management. *BMJ Open Diabetes Res Care* 2018;**6**:e000554. doi:10.1136/bmjdrc-2018-000554

9 International Diabetes Federation. IDF Diabetes Atlas, 7th edn. Brussels, Belgium: International Diabetes Federation 2015. *International Diabetes Federation. IDF Diabetes Atlas, 7th edn. Brussels, Belgium: International Diabetes Federation, 2015.*

10 Mc Hugh S, O'Mullane M, Perry IJ, *et al.* Barriers to, and facilitators in, introducing integrated

For peer review only - http://bmjopen.bmj.com/site/about/guidelines.xhtml

diabetes care in Ireland: a qualitative study of views in general practice. *BMJ Open* 2013;**3**:e003217. doi:10.1136/bmjopen-2013-003217

11 Kutz TL, Roszhart JM, Hale M, *et al.* Improving comprehensive care for patients with diabetes. *BMJ Open Qual* 2018;**7**:e000101. doi:10.1136/bmjoq-2017-000101

12 Bongaerts BWC, Müssig K, Wens J, *et al.* Effectiveness of chronic care models for the management of type 2 diabetes mellitus in Europe: a systematic review and meta-analysis. *BMJ Open* 2017;**7**:e013076. doi:10.1136/BMJOPEN-2016-013076

13 Lim LL, Lau ESH, Kong APS, *et al.* Aspects of Multicomponent Integrated Care Promote Sustained Improvement in Surrogate Clinical Outcomes: A Systematic Review and Meta-analysis. *Diabetes Care* 2018;**41**:1312–20. doi:10.2337/dc17-2010

14 Dambha-Miller H, Cooper AJM, Kinmonth AL, *et al.* Effect on cardiovascular disease risk factors of interventions to alter consultations between practitioners and patients with type 2 diabetes: A systematic review and meta-analysis of trials in primary care. *Heal Expect* Published Online First: February 2017. doi:10.1111/hex.12546

15 Roland M, Everington S. Tackling the crisis in general practice. *BMJ* 2016;**352**:i942. doi:10.1136/BMJ.I942

16 The King's Fund. Public satisfaction with the NHS and social care in 2017. 2017.

17 Maruthappu M, Sood HS, Keogh B. The NHS Five Year Forward View: implications for clinicians. *BMJ* 2014;**349**:g6518.

18 The King's Fund. The NHS long-term plan explained. 2019.

19 Hobbs FDR, Bankhead C, Mukhtar T, *et al.* Clinical workload in UK primary care: a retrospective analysis of 100 million consultations in England, 2007–14. *Lancet* 2016;**387**:2323–30. doi:10.1016/S0140-6736(16)00620-6

20 L.J. H, T.B. D, Renders CM, *et al.* Interventions to improve the management of diabetes mellitus in primary health care and outpatient community settings. L.J. Hansen, Central Forskningsenhed for Almen Praksis og Afdeling for Almen Medicin, Panum Instituttet, Kobenhavns Universitet, DK-2200 Kobenhavn N.: : John Wiley & Sons, Ltd 2002. doi:10.1002/14651858.CD001481

21 Griffin SJ, Kinmonth A-L, Veltman MWM, *et al.* Effect on health-related outcomes of interventions to alter the interaction between patients and practitioners: a systematic review of trials. *Ann Fam Med* 2004;**2**:595–608. doi:10.1370/afm.142

22 Jordan H, Roderick P, Martin D. The Index of Multiple Deprivation 2000 and accessibility effects on health. *J Epidemiol Community Health* 2004;**58**:250–7. doi:10.1136/jech.2003.013011

23 Clegg A, Bates C, Young J, *et al.* Development and validation of an electronic frailty index using routine primary care electronic health record data. *Age Ageing* 2016;**45**:353–60. doi:10.1093/ageing/afw039

24 Turrell G, Oldenburg BF, Harris E, *et al.* Utilisation of general practitioner services by socio-economic disadvantage and geographic remoteness. *Aust N Z J Public Health* 2004;**28**:152–8. doi:10.1111/j.1467-842X.2004.tb00929.x

25 Benchimol EI, Smeeth L, Guttmann A, *et al.* The REporting of studies Conducted using Observational Routinely-collected health Data (RECORD) Statement. *PLoS Med* 2015;**12**:1001885.

For peer review only

For peer review only - http://bmjopen.bmj.com/site/about/guidelines.xhtml

doi:10.1371/journal.pmed.1001885

26 Mathur R, Bhaskaran K, Chaturvedi N, *et al.* Completeness and usability of ethnicity data in UK-based primary care and hospital databases. *J Public Heal (United Kingdom)* 2014;**36**:684–92. doi:10.1093/pubmed/fdt116

27 Lay-Flurrie S, Mathieu E, Bankhead C, *et al.* Patient consultation rate and clinical and NHS outcomes: A cross-sectional analysis of English primary care data from 2.7 million patients in 238 practices. *BMC Health Serv Res* 2019;**19**. doi:10.1186/s12913-019-4036-y

28 Dambha-Miller H, Griffin SJ, Kinmonth AL, *et al.* Provision of services in primary care for type 2 diabetes: a qualitative study with patients, GPs, and nurses in the East of England. *Br J Gen Pract* 2020;**70**:E668–75. doi:10.3399/bjgp20X710945

29 Leszczensky L, Wolbring T. How to Deal With Reverse Causality Using Panel Data? Recommendations for Researchers Based on a Simulation Study. *Sociol Methods Res* 2019;:004912411988247. doi:10.1177/0049124119882473

30 Damen JAAG, Hooft L, Schuit E, *et al.* Prediction models for cardiovascular disease risk in the general population: Systematic review. BMJ. 2016;**353**:i2416. doi:10.1136/bmj.i2416

31 Nunes BP, Flores TR, Mielke GI, *et al.* Multimorbidity and mortality in older adults: A systematic review and meta-analysis. Arch. Gerontol. Geriatr. 2016;**67**:130–8. doi:10.1016/j.archger.2016.07.008

32 Burney P, Jithoo A, Kato B, *et al.* Chronic obstructive pulmonary disease mortality and prevalence: The associations with smoking and poverty-A bold analysis. *Thorax* 2014;**69**:465–73. doi:10.1136/thoraxjnl-2013-204460

For peer review only

Table 1: Summary sociodemographic and clinical characteristics of the Care and Health Information Analytics type 2 diabetes cohort, comprising all patients diagnosed with type 2 diabetes in a linked primary and secondary care electronic database in Hampshire, UK (n = 110,240).

Sex		Patients Missing Data n (%)
Female n(%)	48706 (44.18)	0 (0)
Male n(%)	61534 (55.82)	
Age (years)		
Age	62.89 (14.29)	0 (0)
Ethnicity		
Asian n(%)	3295 (2.99)	54,011 (48.99)
Black n(%)	498 (0.45)	
Mixed or Other n(%)	810 (0.73)	
White n(%)	51626 (46.83)	
Index of Multiple Deprivation		
1 n(%)	14035 (12.73)	1075 (0.98)
2 n(%)	21833 (19.8)	
3 n(%)	21064 (19.11)	
4 n(%)	23062 (20.92)	
5 n(%)	29171 (26.46)	
Smoking Status		
Current Smoker n(%)	13875 (12.59)	492 (0.45)
Ex Smoker n(%)	44875 (40.71)	
Never Smoked n(%)	50998 (46.26)	
Total co-morbidities		
Total co-morbidities	2.36 (1.79)	0 (0)
Individual co-morbidities		
Stroke n(%)	7694 (6.98)	0 (0)

Myocardial Infarction n(%)	9812 (8.9)	0 (0)
Peripheral Vascular Disease n(%)	3565 (3.23)	0 (0)
Chronic Kidney Disease n(%)	15471 (14.03)	0 (0)
Dementia n(%)	3558 (3.23)	0 (0)
Total Medications Prescribed in study period (	48.59 (30.12)	0 (0)
Frailty Score	0.22 (0.11)	77 (0.07)
Values are presented as mean (standard deviation) unless otherwise stated, all values are taken at baseline. HbA_{1c} = glycated haemoglobin		

Table 2: Sociodemographic and clinical characteristics of the Care and Health Information Analytics type 2 diabetes cohort, presented by primary care utilisation tertiles, comprising all patients diagnosed with type 2 diabetes in a linked primary and secondary care electronic database in Hampshire, UK. (n = 75,701).

**Variables**	**All Participants**	**Tertile 1 (low usage)**	**Tertile 2**	**Tertile 3 (high usage)**	**p-value**
Complete case cohort					
Number of patients (n)	75701 (0)	25234 (0)	25234 (0)	25233 (0)	
Average attendances mean (SD)	74.04 (44)	38.6 (8.49)	63.8 (7.87)	119.73 (47.17)	<0.01
Sex					
Female n(%)	34887 (46.09)	9959 (39.47)	11739 (46.52)	13189 (52.27)	<0.01
Male n(%)	40814 (53.91)	15275 (60.53)	13495 (53.48)	12044 (47.73)	<0.01
Age at start (years)					
Age mean(SD)	63.15 (13.04)	60.2 (12.78)	63.05 (12.77)	66.19 (12.87)	<0.01
Age group at start (years)					
18-44 years n(%)	6506 (8.59)	2770 (10.98)	2102 (8.33)	1634 (6.48)	<0.01
45-64 years n(%)	31054 (41.02)	12436 (49.28)	10491 (41.57)	8127 (32.21)	<0.01
65-84 years n(%)	35804 (47.3)	9510 (37.69)	11931 (47.28)	14363 (56.92)	<0.01

For peer review only - http://bmjopen.bmj.com/site/about/guidelines.xhtml

85+ years n(%)	2337 (3.09)	518 (2.05)	710 (2.81)	1109 (4.4)	<0.01
Ethnicity					
Asian n(%)	2097 (2.77)	730 (2.89)	841 (3.33)	526 (2.08)	<0.01
Black n(%)	309 (0.41)	110 (0.44)	118 (0.47)	81 (0.32)	0.03
Mixed or Other n(%)	544 (0.72)	195 (0.77)	182 (0.72)	167 (0.66)	0.34
White n(%)	39519 (52.2)	11272 (44.67)	13543 (53.67)	14704 (58.27)	<0.01
Missing Data n(%)	33232 (43.9)	12927 (51.23)	10550 (41.81)	9755 (38.66)	<0.01
Index of Multiple Deprivation Quintile					
1 n(%)	9301 (12.29)	3311 (13.12)	3013 (11.94)	2977 (11.8)	<0.01
2 n(%)	14215 (18.78)	4857 (19.25)	4621 (18.31)	4737 (18.77)	0.01
3 n(%)	13924 (18.39)	4889 (19.37)	4477 (17.74)	4558 (18.06)	0.56
4 n(%)	16285 (21.51)	5415 (21.46)	5560 (22.03)	5310 (21.04)	0.01
5 n(%)	21368 (28.23)	6612 (26.2)	7348 (29.12)	7408 (29.36)	0.03
Missing IMD Values n(%)	608 (0.8)	150 (0.59)	215 (0.85)	243 (0.96)	<0.01
HbA1c (%)					
HbA1c mean(SD)	7.55 (1.53)	7.43 (1.48)	7.51 (1.49)	7.65 (1.59)	<0.01
HbA1c (mmol/mol)					
HbA1c mean(SD)	59.1 (16.97)	57.85 (16.38)	58.73 (16.49)	60.21 (17.65)	<0.01
BMI (kg)/m^2					
BMI mean(SD)	31.9 (7.31)	31.2 (7.21)	31.1 (7.19)	32.2 (7.38)	<0.01
Electronic frailty index score					
Mean Electronic frailty index score (SD)	0.22 (0.10)	0.16 (0.08)	0.22 (0.09)	0.30 (0.11)	<0.001
Number of co-morbidities					
<= 2 n(%)	40400 (53.37)	18048 (71.52)	13659 (54.13)	8693 (34.45)	<0.01
3-4 n(%)	23970 (31.66)	5996 (23.76)	8575 (33.98)	9399 (37.25)	<0.01
>=5 n(%)	11331 (14.97)	1190 (4.72)	3000 (11.89)	7141 (28.3)	<0.01
Total number of medications prescribed					
<=2 n(%)	1078 (1.42)	737 (2.92)	235 (0.93)	106 (0.42)	<0.01
3-5 n(%)	1037 (1.37)	674 (2.67)	285 (1.13)	78 (0.31)	<0.01

6-9 n(%)	1498 (1.98)	893 (3.54)	419 (1.66)	186 (0.74)	<0.01
>=10 n(%)	72088 (95.23)	22930 (90.87)	24295 (96.28)	24863 (98.53)	<0.01

Data sourced from CHIA dataset Values are presented as mean (standard deviation) unless otherwise stated, all values are taken at baseline. HbA_{1c} = glycated haemoglobin)

For peer review only

Table 3: Univariate (unadjusted) and multivariate (adjusted) models describing the association between primary care service utilisation, defined as number of contacts with primary care services, cardiovascular disease and mortality, in the CHIA data set between 2013 and 2020

	**Unadjusted odds ratio (95% CI) per additional primary care contact**	**Univariate P Value**	**Adjusted** odds ratio (95% CI) per additional primary care contact**	**Multivariate P value**
**Cardiovascular disease***	1.0071 (1.0066 - 1.0074)	<0.001	1.0057 (1.0052 - 1.0061)	<0.001
**Mortality**	1.0077 (1.0070 - 1.0082)	<0.001	1.0056 (1.0050 - 1.0063)	<0.001

** Adjusted for age, sex, ethnicity, index of multiple deprivation, smoking status, number of prescribed medications, frailty, BMI, HbA1c, systolic and diastolic blood pressure, mean total cholesterol, and practice-level co-morbidity prevalence to account for local clustering.

* defined as a composite of myocardial infarction, amputation, and stroke

Figure 1: Mean number of primary care attendances by year quarter (Q1-Q4) between 2013 and 2020 in the CHIA type 2 diabetes cohort. Q1 is defined as January – March.

For peer review only - http://bmjopen.bmj.com/site/about/guidelines.xhtml

Figure 2: Rolling four-quarter mean number of primary care attendances by year quarter between 2013 and 2020 in the CHIA type 2 diabetes cohort stratified by (A) age band (in years) at study entry and (B) ethnicity. Q1 is defined as January – March. For example, the mean number of attendances for Q1 2014 represents mean attendance between Q1 2013 and Q1 2014.

For peer review only

For peer review only - http://bmjopen.bmj.com/site/about/guidelines.xhtml

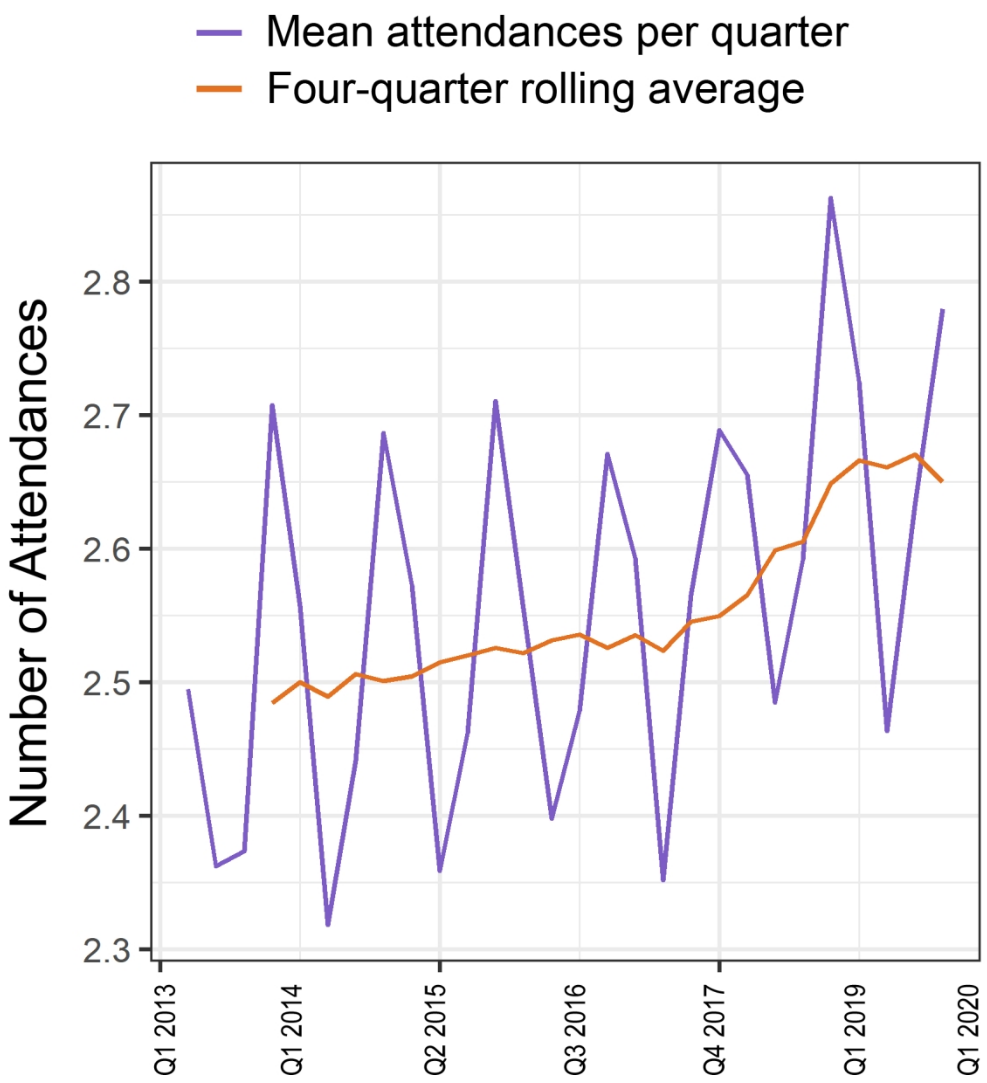

Figure 1: Mean number of primary care attendances by year quarter (Q1-Q4) between 2013 and 2020 in the CHIA type 2 diabetes cohort. Q1 is defined as January – March.

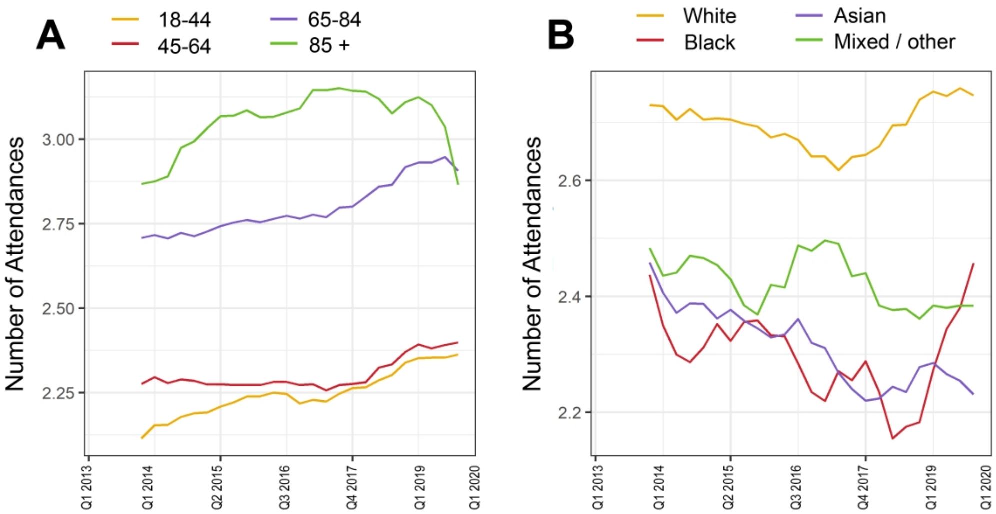

Caption : Figure 2AB: Rolling four-quarter mean number of primary care attendances by year quarter between 2013 and 2020 in the CHIA type 2 diabetes cohort stratified by (A) age band (in years) at study entry and (B) ethnicity. Q1 is defined as January – March. For example, the mean number of attendances for Q1 2014 represents mean attendance between Q1 2013 and Q1 2014.

768x394mm (118 x 118 DPI)

Supplementary File 1: Table to show diagnostic read codes used to identify individuals with type 2 diabetes to include in analysis

**Read Code**	**Condition**
C10F.00	Type 2 diabetes mellitus
C10F000	Type 2 diabetes mellitus with renal complications
C10F011	Type II diabetes mellitus with renal complications
C10F100	Type 2 diabetes mellitus with ophthalmic complications
C10F.11	Type II diabetes mellitus
C10F111	Type II diabetes mellitus with ophthalmic complications
C10F200	Type 2 diabetes mellitus with neurological complications
C10F211	Type II diabetes mellitus with neurological complications
C10F300	Type 2 diabetes mellitus with multiple complications
C10F311	Type II diabetes mellitus with multiple complications
C10F400	Type 2 diabetes mellitus with ulcer
C10F411	Type II diabetes mellitus with ulcer
C10F500	Type 2 diabetes mellitus with gangrene
C10F511	Type II diabetes mellitus with gangrene
C10F600	Type 2 diabetes mellitus with retinopathy
C10F611	Type II diabetes mellitus with retinopathy
C10F700	Type 2 diabetes mellitus - poor control
C10F711	Type II diabetes mellitus - poor control
C10F800	Reaven's syndrome
C10F811	Metabolic syndrome X
C10F900	Type 2 diabetes mellitus without complication

For peer review only

C10F911	Type II diabetes mellitus without complication
C10FA00	Type 2 diabetes mellitus with mononeuropathy
C10FA11	Type II diabetes mellitus with mononeuropathy
C10FB00	Type 2 diabetes mellitus with polyneuropathy
C10FB11	Type II diabetes mellitus with polyneuropathy
C10FC00	Type 2 diabetes mellitus with nephropathy
C10FC11	Type II diabetes mellitus with nephropathy
C10FD00	Type 2 diabetes mellitus with hypoglycaemic coma
C10FD11	Type II diabetes mellitus with hypoglycaemic coma
C10FE00	Type 2 diabetes mellitus with diabetic cataract
C10FE11	Type II diabetes mellitus with diabetic cataract
C10FF00	Type 2 diabetes mellitus with peripheral angiopathy
C10FG00	Type 2 diabetes mellitus with arthropathy
C10FG11	Type II diabetes mellitus with arthropathy
C10FH00	Type 2 diabetes mellitus with neuropathic arthropathy
C10FJ00	Insulin treated Type 2 diabetes mellitus
C10FJ11	Insulin treated Type II diabetes mellitus
C10FK00	Hyperosmolar non-ketotic state in type 2 diabetes mellitus
C10FL00	Type 2 diabetes mellitus with persistent proteinuria
C10FL11	Type II diabetes mellitus with persistent proteinuria
C10FM00	Type 2 diabetes mellitus with persistent microalbuminuria
C10FM11	Type II diabetes mellitus with persistent microalbuminuria
C10FN00	Type 2 diabetes mellitus with ketoacidosis

For peer review only

C10FP00	Type 2 diabetes mellitus with ketoacidotic coma
C10FQ00	Type 2 diabetes mellitus with exudative maculopathy
C10FR00	Type 2 diabetes mellitus with gastroparesis

For peer review only
